# Senolytic treatment reduces oxidative protein stress in an aging male murine model of post‐traumatic osteoarthritis

**DOI:** 10.1111/acel.13979

**Published:** 2023-09-25

**Authors:** Alexander F. Chin, Jin Han, Cristina C. Clement, Younghwan Choi, Hong Zhang, Maria Browne, Ok Hee Jeon, Jennifer H. Elisseeff

**Affiliations:** ^1^ Translational Tissue Engineering Center, Wilmer Eye Institute and Department of Biomedical Engineering Johns Hopkins University School of Medicine Baltimore Maryland USA; ^2^ Department of Radiation Oncology Englander Institute for Precision Medicine, Weill Cornell Medicine New York New York USA; ^3^ Department of Biomedical Sciences Korea University College of Medicine Seoul Republic of Korea; ^4^ Bloomberg‐Kimmel Institute for Cancer Immunotherapy, Johns Hopkins University School of Medicine Baltimore Maryland USA

**Keywords:** aging, cellular senescence, mouse, osteoarthritis, oxidative stress, protein chemistry, proteomics, reactive oxygen

## Abstract

Senolytic drugs are designed to selectively clear senescent cells (SnCs) that accumulate with injury or aging. In a mouse model of osteoarthritis (OA), senolysis yields a pro‐regenerative response, but the therapeutic benefit is reduced in aged mice. Increased oxidative stress is a hallmark of advanced age. Therefore, here we investigate whether senolytic treatment differentially affects joint oxidative load in young and aged animals. We find that senolysis by a p53/MDM2 interaction inhibitor, UBX0101, reduces protein oxidative modification in the aged arthritic knee joint. Mass spectrometry coupled with protein interaction network analysis and biophysical stability prediction of extracted joint proteins revealed divergent responses to senolysis between young and aged animals, broadly suggesting that knee regeneration and cellular stress programs are contrarily poised to respond as a function of age. These opposing responses include differing signatures of protein‐by‐protein oxidative modification and abundance change, disparate quantitative trends in modified protein network centrality, and contrasting patterns of oxidation‐induced folding free energy perturbation between young and old. We develop a composite sensitivity score to identify specific key proteins in the proteomes of aged osteoarthritic joints, thereby nominating prospective therapeutic targets to complement senolytics.

## Graphical abstract

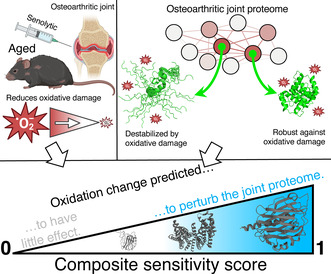

Senolytic treatment by a p53/MDM2 interaction inhibitor reduces joint protein oxidative stress load in an aged mouse model of osteoarthritis (OA). Mass spectrometry proteomic interrogation paired with protein interaction network and biophysical stability analysis reveals divergent responses to senolysis between young and aged animals. We develop a composite sensitivity score to identify specific key proteins in the proteomes of aged osteoarthritic joints.

**Abbreviations**
%Δ PTM/site: percent change in post‐translational modification per post‐translational modification site
4‐ONE: 4‐oxononenal
ACLT: anterior cruciate ligament transection
AGE: advanced glycation end product
ALE: advanced lipoxidation end product
ANOVA: analysis of variance
CSS: composite sensitivity score
ECM: extracellular matrix
ER: endoplasmic reticulum
G‐H1: glyoxal‐derived hydroimidazolone
GO: gene ontology
HNE: 4‐hydroxynonenal
LFQ: label‐free quantification
MMP: matrix metalloproteinase
MS: mass spectrometry
OA: osteoarthritis
PTM: post‐translational modification
PTOA: post‐traumatic osteoarthritis
ROS: reactive oxygen species
SASP: senescence associated secretory phenotype
SnC: senescent cell

## 1 INTRODUCTION

Aging mammalian tissues accumulate senescent cells (SnCs), a population of cells that have stably exited the cell cycle and entered a non‐replicative state (van Deursen, 2014). Once established, SnCs actively emit a cocktail of signaling molecules that shape the local tissue environment. While in some cases SnCs can be cleared by the immune system, chronic loads of SnCs in tissues can establish entrenched, inflammatory milieus. The complex mixture of paracrine signals released by SnCs is termed the senescence associated secretory phenotype (SASP) (Coppé et al., 2008). Selective killing of SnCs in mice via genetically engineered constructs qualitatively rejuvenates aged animals by reducing the severity of age‐related pathology and extending lifespan, likely in part due to the elimination of chronic SASP exposure in tissues (Baker et al., 2016). A translational alternative to genetically engineered SnC kill‐switches are therapeutic molecules, termed senolytics, which promote SnC killing often by disrupting anti‐apoptotic pathways otherwise highly upregulated in SnCs (Lozano‐Torres et al., 2019). A variety of senolytics are being advanced to clinical trials (Dolgin, 2020).

More recently, senolytics have been deployed to treat age‐ and injury‐related pathologies, where efforts previously focused on pharmacological, surgical or traditional biomaterial engineering solutions. Osteoarthritis (OA) is an example of one such application. OA of the synovial joints disproportionally afflicts older individuals and represents a medical need with outsize economic consequences (Hunter et al., 2014). Traumatic injury of the knee predisposes individuals to developing OA (Lohmander et al., 2007). In a mouse model of post‐traumatic OA (PTOA), traumatic anterior cruciate ligament transection (ACLT) injury promotes the accumulation of SnCs in the knee joint tissue (Jeon et al., 2017). Subsequent removal of these SnCs through senolytic dosing reduced injury associated pain, reduced expression of inflammatory markers, and increased expression of prochrondrogenic markers. In contrast, when similar treatment was administered to aged mice, injury associated pain and inflammatory markers were reduced, but prochrondrogenic markers remained unchanged. Additional systemic senolytic administration was required in aged animals to increase treatment effectiveness, revealing a treatment barrier born from the synergistic interplay between age and injury (Faust et al., 2020).

The interaction between senolytic efficacy and age led us to ask whether a confounding factor could explain the reduced osteoarthritic treatment benefit. Could an alternative, relevant physiological process underlying aging be responsible? We considered oxidation, specifically metabolic redox stress mediated protein oxidation, as one such salient process. The steady accumulation of cellular oxidative damage is a broadly shared feature of biological aging (Stadtman, 2006). Free reactive oxygen species (ROS) produced by metabolism and respiration oxidize proteins, lipids, nucleic acids, or other cellular components, eventually leading to durable covalent modification and functional impairment. The presence of oxidized protein products in the joint have been shown to enhance OA pathology and the expression of SASP factors, such as matrix metalloproteinase (MMP)3 and MMP13 (Yu et al., 2015). More generally, oxidative stress in the joint has been shown to contribute to chondrocyte apoptosis and senescence, synovial inflammation, and cartilage damage (Lepetsos & Papavassiliou, 2016). ROS have been observed to be released from mechanically stressed chondrocytes, thus plausibly positioning mechanical injury as a driver of joint oxidative stress (Martin et al., 2004).

In this study, we explored whether senolytics can abrogate oxidative stress in the aged, osteoarthritic, mouse knee joint. We found that senolytic treatment diminished the amount of protein oxidative post‐translational modifications (PTMs) in aged mouse knees. Unexpectedly, senolytic treatment yielded not only different, but often opposing effects between young and aged mice, evident in several facets of our data: cartilage protein relative abundance profiles, quantitative patterns of protein oxidative PTMs, the cartilage protein–protein interaction network, and an integrative biophysical model that relates protein oxidation to protein stability. Finally, we developed a composite sensitivity score to identify specific protein targets in the osteoarthritic aging knee proteome that may be therapeutically leveraged to complement senolytic effectiveness in advanced age.

## 2 RESULTS

**FIGURE 1:**
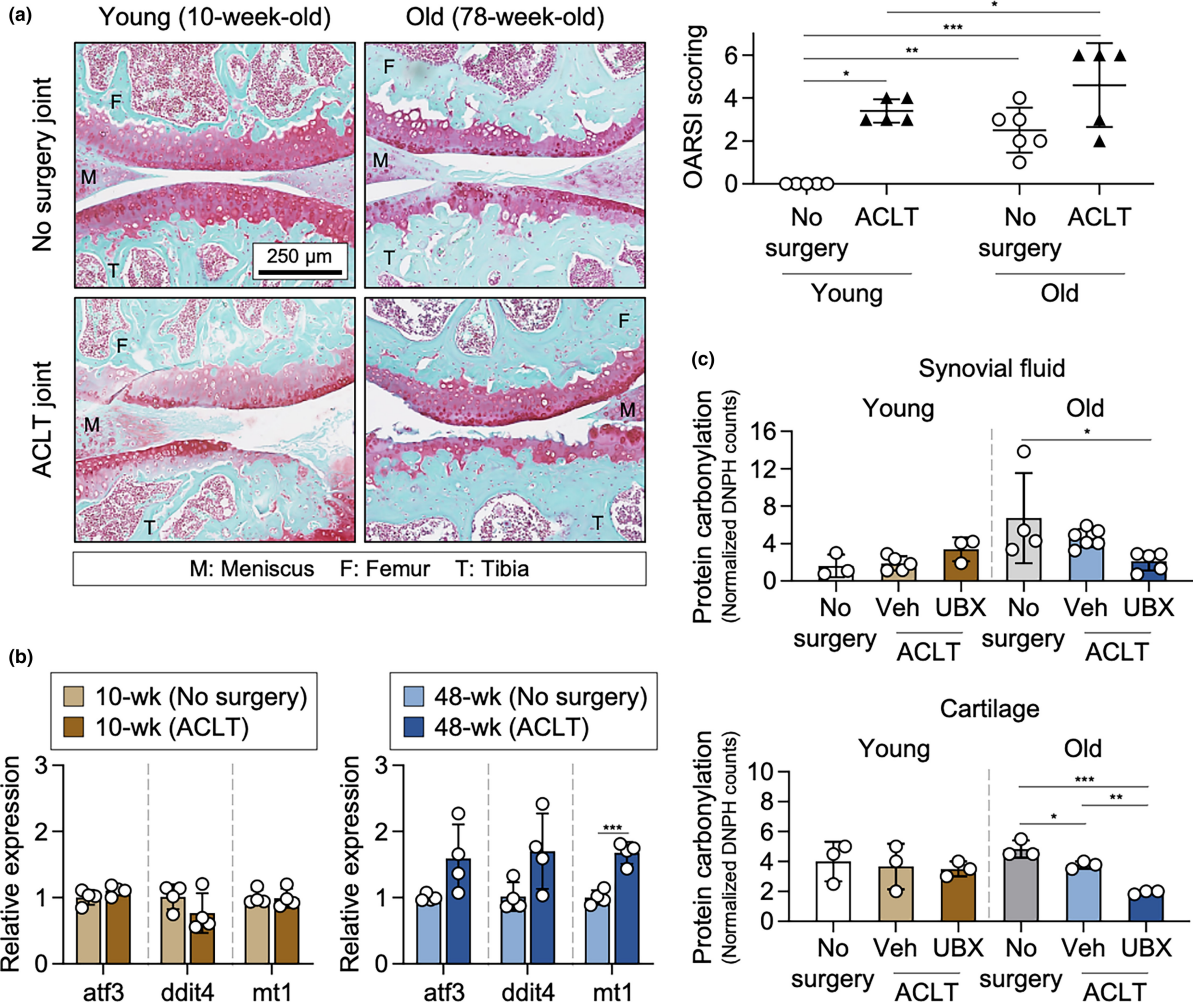
Senolytic treatment reduces the extent of protein oxidative modification in osteoarthritic joints of aged mice. (a) Images (left) and quantification (right) of Safranin‐O histological stains of the mouse knee joint comparing young and old mice with or without ACLT surgery. *n* = 5–6 biologically independent samples, two‐way ANOVA with Tukey's multiple‐comparisons test. (b) Quantification of genes associated with stress response and oxidative stress in young and aged animals 4 weeks after ACLT injury. *n* = 4 biologically independent samples, one‐way ANOVA with Tukey's multiple‐comparisons test within the relative age groups. (c) Quantification of anti‐DNPH intensity between young and old animals after injury and treatment in synovial fluid (top) and cartilage (bottom). *n* = 3 biologically independent samples. *p < 0.05, **p < 0.01, and ***p < 0.001. For all bar graphs, data are mean ± SD.

To interrogate the effect of senolytics on oxidative stress in aged, OA mice, we first performed ACLT injury surgeries on variously aged male mice to induce OA. ACLT injury surgery performed on mice of advanced age was previously shown to stimulate production of SnC throughout the knee articular cartilage as well as induce OA (Jeon et al., 2017). Safranin‐O histological staining of proteoglycans, a commonly used method to assess osteoarthritic progression, revealed cartilage degradation as a baseline feature of old age without ACLT, as well as a more severe post‐ACLT OA pathology in older mice (Figure 1a) (Pauli et al., 2011). However, histological staining alone cannot reveal whether heightened oxidative stress develops in tandem with structural declines. We therefore used RT‐qPCR to probe gene expression markers broadly associated with oxidative stress in the knee cartilage, finding signs of oxidative imbalance in 48‐week‐old middle‐aged mice compared to 10‐week‐old young mice (Figure 1b). The oxidative stress expression signature consistently trended towards an increase in stress after ACLT in increasing age, though not all markers reached the threshold of statistical significance. We reasoned that these temporal snapshots suggested a dynamic of progressive age‐dependent decline, and predicted a more pronounced effect size would be observed in more advanced age. As a result, we subsequently used 78‐week/20‐month‐old aged mice for further experiments to probe the effect of ACLT and the senolytic drug UBX0101, a p53/MDM2 interaction inhibitor, on the joint oxidative environment. An exploratory in vivo assessment using an ROS‐sensitive fluorescent reporter molecule in 78‐week aged mouse knees hinted at a capability of UBX0101 to modulate the local knee oxidative milieu (Figure S1).

While a variety of cellular molecules may serve as ROS sources or sinks, we chose to focus on oxidative response in the protein component of the aged OA joint as a function of ACLT and senolytic treatment. Along these lines, carbonylation assays were performed on extracted synovial fluid and cartilage proteins, where carbonylated protein groups were chemically derivatized and detected by western blot (Figure S1). The degree of carbonylation reflects the overall quantity of protein oxidative stress products. We observed that UBX0101‐treated ACLT aged mice had a significantly reduced quantity of carbonylated protein compared to no‐surgery controls, both in the synovial fluid and in the cartilage (Figure 1c). Taken together, these transcription and protein‐based data broadly correlate to suggest a correspondence of advancing age and increasing joint oxidation, which can be modified by the administration of the senolytic UBX0101.

**FIGURE 2:**
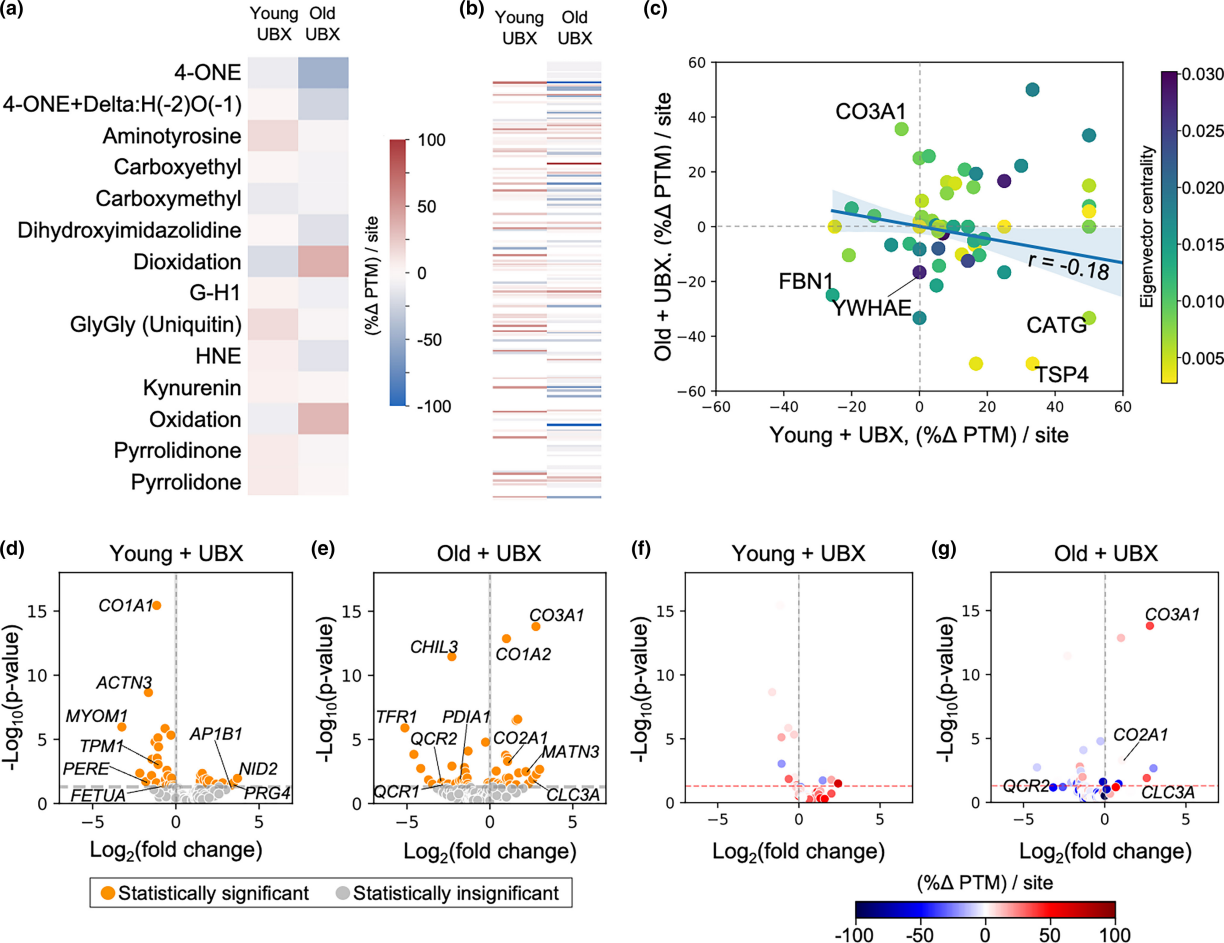
Senolytic treatment is associated with an overall decrease of oxidative PTMs in OA cartilage of aged mice, as well as divergent, age‐dependent proteomic landscapes (a) Heatmap representing MS‐detected PTM changes in young and old OA mice after treatment with the senolytic UBX0101, grouped by chemical identity of the PTM. (b) Heatmap representing MS‐detected PTM changes in young and old OA mice after treatment with the senolytic UBX0101, grouped by protein. Each row represents one protein. Two hundred forty‐three unique proteins are represented. The color bar legend is shared between heatmaps. (c) Scatterplot relating MS‐detected PTM changes after treatment with the senolytic UBX0101 in young and old OA mice. Each point represents one protein. The solid line represents a linear regression model fit, Pearson's *r* = −0.18. The translucent band shows the 95% bootstrap confidence interval for the regression. (d) Young mouse and (e) old mouse label‐free quantification generated by mass spectrometric analysis. The horizontal dotted line indicates the threshold of statistical significance at *p* < 0.05. (f) MS‐detected PTM changes in young OA mice and (g) in old OA mice after treatment with the senolytic UBX0101, graphically overlaid on LFQ values, on a per‐protein basis. In addition to no change in PTM status, the color white may also represent insufficient data to calculate a PTM change.

With this in mind, we interrogated the molecular details of the knee cartilage landscape using mass spectrometry (MS) and label‐free quantitation (LFQ) analysis to detect oxidation‐associated, covalent, post‐translational protein modifications in young and old OA mice with or without senolytic treatment (Figure S1). The input material consisted of extracted joint cartilage, which we estimated did not contain confounding quantities of non‐cartilage contamination (Figure S2). Our proteomic survey included a battery of PTMs described as advanced glycation and lipoxidation end products (AGEs and ALEs, respectively), whose accumulation are known to robustly track with age and age‐associated diseases (Chaudhuri et al., 2018). AGEs and ALEs are the result of non‐enzymatic reactions of protein with reducing sugars, or with oxidized sugar or lipid degradation products, which may alter or crosslink the positively charged amino acids lysine and arginine on proteins (Vistoli et al., 2013). Our dataset supported detection of several AGEs and ALEs, including the adducts 4‐ONE (4‐oxononenal), 4‐ONE+Delta:H(−2)O(−1) (dehydrated 4‐oxononenal Michael adduct), carboxyethyl, carboxymethyl, 3‐deoxyglucosone derived dihydroxyimidazoline, G‐H1 (glyoxal‐derived hydroimidazolone), HNE (4‐hydroxynonenal), and several carbonylated adducts including proline to pyrrolidinone or pyrrolidone (Figure 2a). Other detected modifications of note include mono‐oxidation and di‐oxidation products, including tryptophan to kynurenin (Berlett & Stadtman, 1997). We also detected lysine‐epsilon‐gly‐gly (GlyGly), which marks ubiquitylated sites, indicating potential substrates of proteasome‐dependent degradation (Fulzele & Bennett, 2018).

Aged, OA mice treated with senolytic displayed a pattern of greater breadth and magnitude of oxidation‐associated PTM reduction when compared to treated young OA mice (Figure 2a,b). PTM changes were determined as the percent change in PTM per PTM site (%Δ PTM/site) when senolytic‐treated tissue was compared to vehicle treated tissue, a metric chosen to enable normalized comparison between proteins. The metric was then summed either across modifications or across individual proteins (Figure 2a,b; Figure S3). The grand sum PTM change was negative for treated aged OA mice but positive for treated young OA mice, regardless of whether summed across modifications or across proteins. In other words, senolytic treatment reduced oxidation‐associated PTMs more effectively in aged OA mice than in young OA mice. This result corroborated our above observations of reduced protein bulk carbonylation in the UBX0101 treated joint. On a PTM chemical species basis, most AGEs/ALEs identified by our bottom‐up proteomics assay decreased in abundance in senolytic‐treated aged OA animals (10 out of 14 modifications in treated aged OA animals, 5 of 14 modifications in treated young OA animals), suggesting a broader, more generalized contraction of the AGE/ALE proteomic modification profile in old age. Of the three modifications where decrease of AGE/ALEs agreed in sign between senolytic‐treated young and aged OA animals, the response magnitude in aged OA animals was greater in two of those three cases (4‐ONE: −47% old vs. −11% young; 3‐deoxyglucosone derived dihydroxyimidazoline: −17% old, −1% young; carboxymethyl: −7% old, −13% young). A similar pattern emerged when the PTM changes were examined on a per‐protein basis. A greater variety of unique proteins in senolytic‐treated aged OA mice were observed to decrease in assessed PTMs (73 decreasing, 30 increasing), compared to senolytic‐treated young OA mice (12 decreasing, 51 increasing).

A gene ontology (GO) enrichment analysis was applied to the proteins with the greatest decreases in percent PTM per site (Table S1) (The Gene Ontology Consortium, 2019). Extracellular matrix (ECM) components were significantly overrepresented, as were cytoskeletal or cell motility components. Of those that had reduced PTMs, several notable examples included proteins indicative of OA severity, a protease of the joint lubricator protein lubricin, and osteoblast differentiation modulators.

In addition to comparing the grand sum PTM change between young and old senolytic treatment, we computed the quantitative relationship of %Δ PTM per site on a per‐protein basis between young and old senolytic‐treated animals (Figure 2c). We found that a modest anti‐correlation predicts an age‐dependent opposing response to senolytic treatment. In other words, the trend in both sign and degree of per‐protein %Δ PTM per site reinforces our observations where young and aged senolytic response are not only dissimilar, but divergent.

Having established that senolytic treatment reduces oxidation‐associated PTMs with efficacy in aged OA mice, we asked whether shifts in protein levels might further explain differential responses to senolytic between young and old OA animals. Relative protein abundance changes upon senolytic treatment revealed differing patterns between young and old mice. In young OA mice, GO analysis identified upregulation of extracellular components with known roles in the joint or cartilage matrix, and downregulation of cytoskeletal and muscle‐associated elements (Figure 2d; Table S2). Overall, relative protein abundance changes are consistent with young OA mice initiating a program of cartilage matrix protein production and chondrocyte differentiation upon senolytic therapy, which is supported by previously reported therapeutic response (Jeon et al., 2017).

As a whole, senolytic‐driven protein abundance shifts in aged OA animals contrast with the shifts observed in young OA animals. (Figure 2e; Table S3). GO analysis of protein changes in aged animals indicated upregulation of collagens and extracellular proteins, while mitochondrial respiratory activity and an alternate set of collagen and extracellular proteins were downregulated. The particular mixture of changes suggested a combination of OA diseased cartilage processes, compensatory OA‐protective alterations in immune signaling capabilities, and adaptation to an oxidative environment. Notable upregulated outlier proteins include the cytosolic molecular chaperone HSP90 and several histones (Table S4).

Two mitochondrial cytochrome bc1 complex subunits and protein disulfide isomerase all downregulated in senolytic‐treated aged OA animals best highlight the contrast between young and aged protein abundance changes. The mitochondrial bc1 complex, also known as complex III, is a component of the respiratory electron transport chain which, importantly, is capable of leaking electrons during normal operation to generate superoxide, an ROS (Dröse & Brandt, 2012). Protein disulfide isomerase is a chaperone enzyme that oxidizes cysteines in disulfide bridges to assist in protein folding as part of the endoplasmic reticulum (ER) unfolded protein response. The activity of protein disulfide isomerase requires electron transfer and use of a finite pool of glutathione, in turn generating ROS as a side product of the unfolded protein response (Bhandary et al., 2013). The coincident reduction of these ROS generating proteins agrees with our experimental results showing that senolytic treatment of aged OA animals reduced oxidative modifications on joint extracted proteins. In sum, aged animals appeared to deploy a mixture of expression programs simultaneously consistent with increased OA‐pathogenic cartilage deposition and proteostatic stress response, but lessened OA‐pathogenic immune signaling triggers and ROS production.

Given the divergent protein abundance profiles of young and aged OA animals, we asked whether the shifts in protein abundance correlated with a pattern of increasing or decreasing oxidatively driven PTMs. Thus, we cross‐referenced PTM percent changes with protein abundance changes, on a per‐protein basis (Figure 2f,g). No clear association between degree or sign of oxidatively driven PTM change and degree or sign of protein abundance was apparent. As a result, we returned to further examine the age‐dependent anti‐correlative trend of per‐protein %Δ PTM per site, reasoning that additional quantitative relationships might be revealed if these data were contextualized within their cellular environment.

**FIGURE 3:**
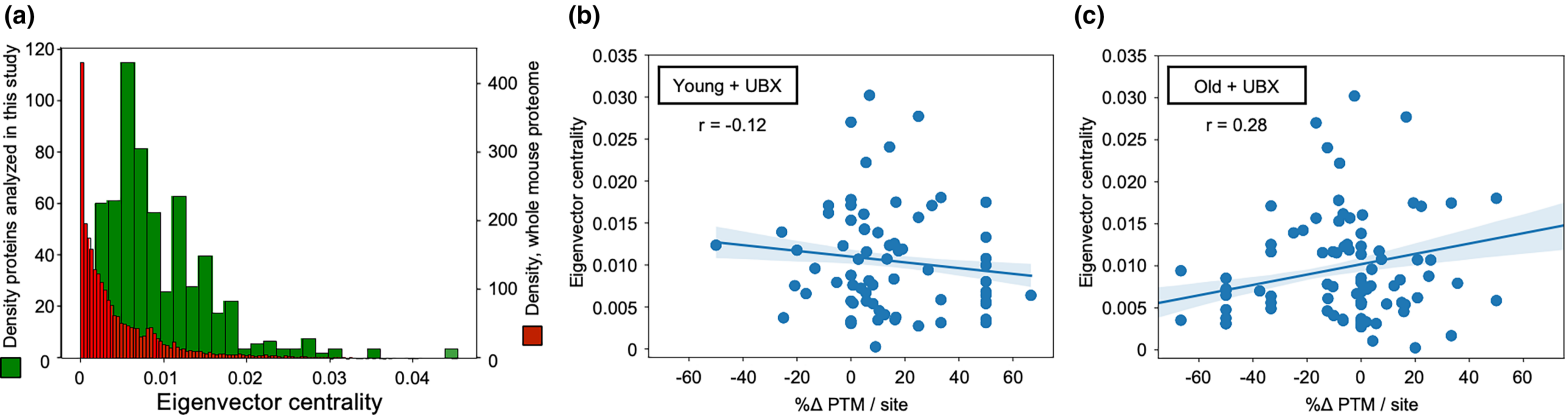
Senolytic‐associated shifts in oxidative PTMs predict age‐dependent, opposing capacities of treatment to influence the OA joint protein–protein interaction network. (a) Empirical probability density histograms of eigenvector centrality representing the entire mouse protein interaction network (21,319 proteins and 7,248,180 interactions) and the subnetwork of OA joint proteins in this study (238 proteins and 6050 interactions). (b) Scatterplot relating eigenvector centrality to MS‐detected PTM changes after treatment with the senolytic UBX0101 in young OA mice (Pearson's *r* = −0.12) and (c) in old OA mice (Pearson's *r* = +0.28). Each point represents one protein. The solid lines represent linear regression model fits. The translucent bands show the 95% bootstrap confidence intervals for the regressions.

To that end, we calculated the eigenvector centrality for each protein in the known mouse protein–protein interaction network, whose network topology was acquired from the STRING database. Eigenvector centrality is a metric that recursively scores the influence of a node in a network, where in this case, a protein is awarded high centrality if it interacts with partner proteins themselves of high centrality, and so on. The MS experimentally detected proteins in this study were defined as a subnetwork for further analysis, such that the eigenvector centralities of that subnetwork remain numerically calibrated to the eigenvector centrality scale of the whole mouse proteome (Figures 2c, 3a; Figure S4). Inspection of the eigenvector centrality distribution shows that, on average, the subnetwork of proteins in this study has somewhat higher network influence than those in the mouse proteome as a whole, but also that the two distributions exhibit a skewed shape, containing few members of the highest centrality (Figure 3a). Superimposition of eigenvector centrality upon the young and old senolytic‐driven %Δ PTM per site relationship reveals anisotropic centrality spread between proteins (Figure 2c). Upon closer inspection, a quantitative relationship emerges when the young and old %Δ PTM per site are examined independently, such that modest but contrary trends in eigenvector centrality versus %Δ PTM per site predict opposite relationships between the young and old senolytic‐treated OA joint (Figure 3b,c). In the young joint, the pattern predicts that oxidative PTM gain occurs on proteins of less influence, while loss occurs on proteins of greater influence. In the old joint, a predictive association, double in relative strength, shows oxidative PTM gain occurs on proteins of greater influence, while loss occurs on proteins of lesser influence. We note that in addition to the stronger correlation in the old condition, a greater absolute count of proteins experience oxidative PTM loss in older mice, potentially reinforcing the impact of the young‐old asymmetry. Taken together, opposing quantitative trends relating %Δ PTM per site to a protein's relative influence within protein–protein interaction networks bolster broader observations of opposite senolytic responses as a function of age.

**FIGURE 4:**
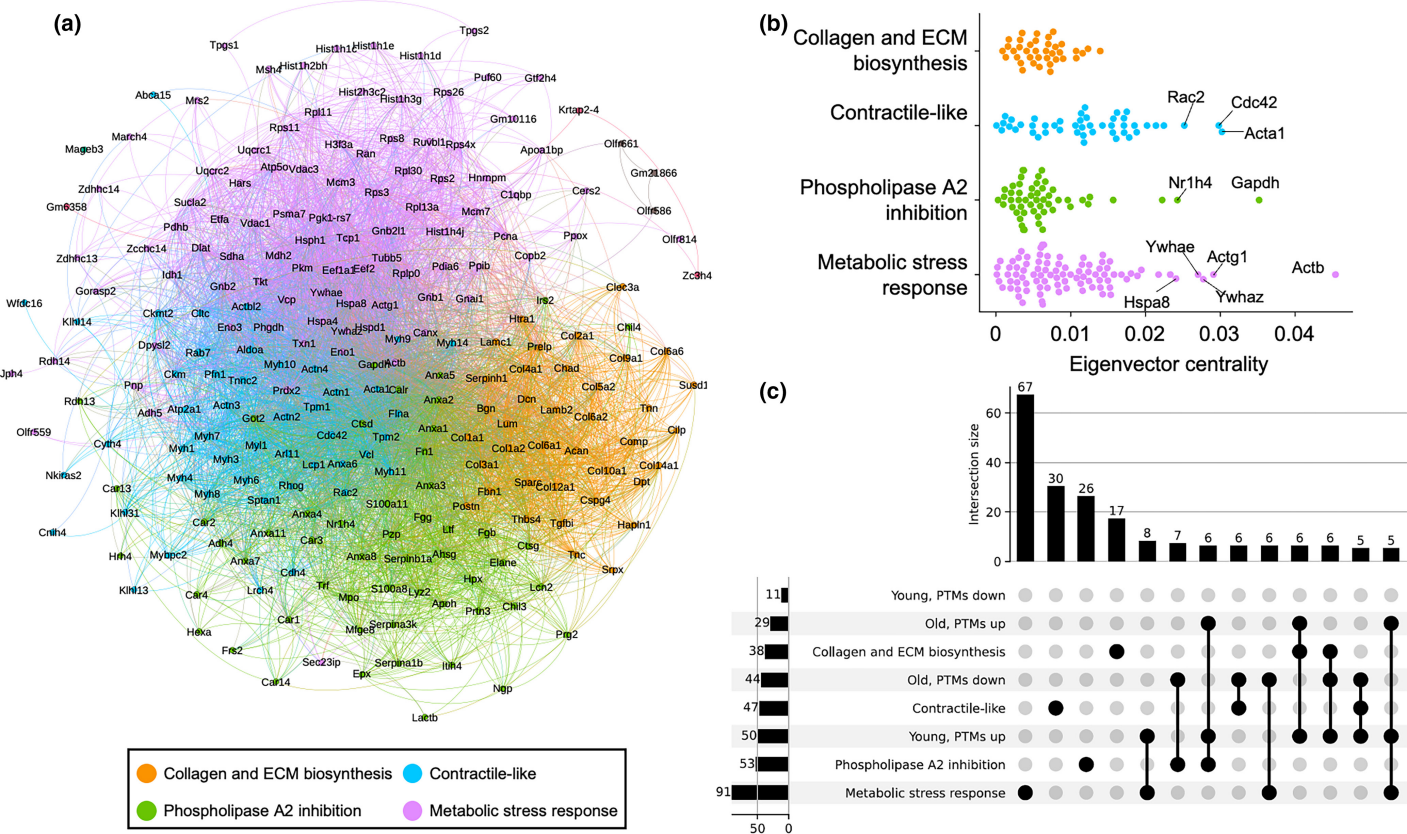
Senolytic‐associated oxidative PTMs affect diverse functional facets of the OA joint proteome. (a) A network graph illustration of the OA joint protein interaction network, colored by the four major network communities detected by the Louvain modularity optimization method. (b) Swarm plot comparing eigenvector centrality distributions between the Louvain‐detected network communities. (c) UpSet plot showing set membership between the given network communities and age‐dependent, senolytic‐associated oxidative PTM changes. Within age variables, PTM changes have been binarily grouped between positive and negative %Δ PTM per site.

We next assigned a qualitative functional description to subgroups in the protein–protein interaction network. To accomplish this, we deployed the Louvain algorithm for network community detection, which optimizes the modularity of protein groups such that the resulting communities are denser within themselves than between each other. The Louvain procedure partitioned the largest connected component of the network into four major communities which we then examined by Reactome pathway and Gene Ontology analysis (Figure 4a; Figure S4). Based on overrepresented pathway and GO terms, we summarized these communities as “Collagen and ECM biosynthesis” (modification of collagen and ECM), “Contractile‐like” (actin‐based motility or cytoskeletal processes), “Phospholipase A2 inhibition” (phospholipase A2 exhibits pro‐inflammatory activity in OA and has been shown to induce senescence) and “Metabolic stress response” (involving translational, energetic, and mitochondrial homeostasis) (Figure 4b, Table S5). The eigenvector centrality distributions of each community shared a similarly skewed shape, though the higher percentiles of eigenvector centrality were mostly confined to the metabolic stress response and contractile‐like groups. When we examined membership of the communities based on the sign of their age‐dependent oxidative PTM changes, we found that although the Louvain communities were successfully well‐defined, no single bipartite or tripartite combination of age, PTM change, and Louvain community had significantly greater membership count than the others (Figure 4c; Figure S5).

We chose to complement our interaction network analysis by invoking a quantitative biophysical model, which enhanced our power to predict whether oxidation of certain proteins could contribute disproportionately to proteomic perturbation. To achieve this, we considered the dependence of a protein's stability on its charge state. Oxidative PTMs, including AGE/ALE, have the capacity to alter protein charge as a function of their chemical identity or through neutralization of charged amino acids such as lysine or arginine, which are typical sites of AGE/ALE adducts. We leveraged an established biophysical model of protein electrostatics that relates the folding free energy change of a protein, that is, its overall stability, to its charge status and length or average size of that protein (De Graff et al., 2016). This model allowed us to calculate a predictive map describing the susceptibility of a given protein's folding stability upon adding or losing a charge altering, oxidative PTM. When the entire human or mouse proteome were mapped on to this oxidative susceptibility space, most proteins clustered in a region of low susceptibility, stemming from low net charge and short length, as other studies have validated (Figure S6) (De Graff et al., 2016). Interestingly, while long‐lived mammals such as the naked mole rat, Brandt's bat, or African elephant showed similar proteomic distributions in oxidative susceptibility space, the high‐susceptibility extremes of the distribution were modestly but consistently less susceptible to oxidative destabilization when compared to the human or mouse proteomes (Figure S6).

**FIGURE 5:**
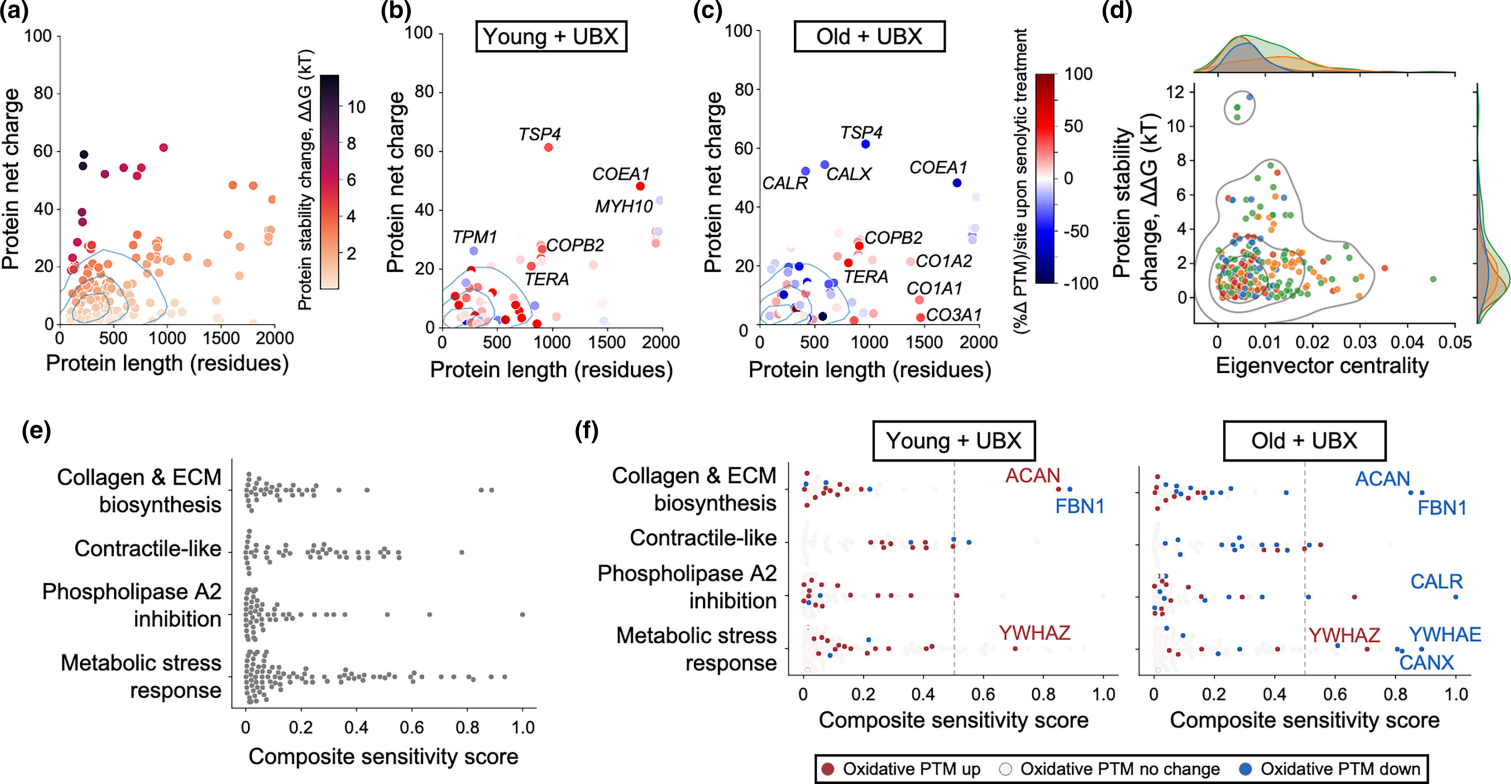
A biophysical model of oxidation‐induced protein destabilization highlights an age‐dependent senolytic‐proteostasis interface, and nominates potential therapeutic targets of the OA joint proteome. (a) Net charge versus protein length diagrams, where blue rings delineate quartiles of an overlaid empirical density mapping of the whole mouse proteome. Proteins in this study are mapped as individual points. Darker colors represent higher potential for destabilization, predicted by modeling hypothetical, electrostatic‐driven protein destabilization caused by a single oxidation event. (b) Senolytic‐treated young OA mice experience mixed oxidative PTM changes in destabilization‐sensitive proteins. Color scale corresponds to the senolytic‐associated %Δ PTM. In addition to no change in PTM status, the color white may also represent insufficient data to calculate a PTM change. (c) Senolytic‐treated aged OA mice experience decreases in oxidative PTMs in destabilization‐sensitive proteins. The color scale is shared with panel B. (d) Sensitivity to destabilization by oxidation‐induced charge change versus eigenvector centrality for proteins represented in this study. A bivariate kernel density estimate is superimposed in gray, while points and joint distributions are colored by network community: blue = collagen and ECM biosynthesis, orange = contractile‐like, red = phospholipase A2 inhibition, green = metabolic stress response. (e) Comparison of composite sensitivity score distributions between network communities. (f) Comparison of composite sensitivity score distributions, color coded with oxidative PTM change directionality in the young and old senolytic‐treated conditions.

To determine the oxidatively driven destabilization sensitivity of the articular joint proteins examined here, we mapped proteins where we detected at least one PTM change in our MS data on to the oxidative susceptibility space (Figure 5a). Overall, the net charge and protein length distributions of the detected proteins track with overall density trends for the complete mouse proteome, with the majority in the low net charge and length region, corresponding to low susceptibility to oxidative stability change. Twenty‐three percent of our detected joint proteins fell into a region corresponding to the highest proteomic quartile of high net charge, long length proteins, corresponding to heightened susceptibility to oxidative stability change.

Using this mapping, we compared the differential effect of senolytic treatment between young and aged OA mice, with attention to those proteins especially prone to destabilization by charge changing, oxidatively driven PTMs (Figure 5b,c; Table S6). We found that several of these susceptible proteins changed their PTM status upon senolytic treatment, but in opposite directions depending on whether they were detected in young or aged OA mice. These include aggrecan, thrombospondin‐4, tropomyosin alpha−1 chain, cathepsin G, and collagen type XIV. In contrast, other susceptible proteins which had changed PTM status upon senolytic treatment, but in similar directions include reduction of PTMs on fibrillin−1, and increase in PTMs on myeloperoxidase, lactotransferrin, and alpha‐actinin−1.

A noteworthy pair of proteins uniquely altered in aged, senolytic‐treated OA animals was calreticulin and calnexin. We observed PTM decrease on both of these high‐susceptibility proteins. Calreticulin and calnexin are endoplasmic reticular chaperones that act on glycoproteins fated for cellular export, ensuring the quality of the final protein released (Bedard et al., 2005). As some of the notable synovial or cartilage proteins are glycoproteins, such as lubricin, cartilage matrix glycoprotein, and fibronectin, among others, the quality control activities of calreticulin and calnexin may have an outsize influence on the functional health of the joint (Burton‐Wurster et al., 1997; Fife, 1988). Along these lines, the PTM decreases on calreticulin and calnexin may specifically interact with the detected oxidatively driven PTM status changes of the glycoproteins aggrecan, thrombospondin‐4, and fibrillin‐1.

Finally, we explored the interplay between oxidation‐induced protein destabilization and eigenvector centrality among the different Louvain communities of the joint cartilage protein–protein interaction network. We reasoned that the proteins where oxidative PTM change would be most impactful in altering the joint proteomic milieu would be a blend of those where stability was highly perturbed and those that were centrally influential in the network. Inspection of the bivariate distribution of stability change and centrality revealed heavier tails of both dimensions in the metabolic stress response community and an overall density of all represented proteins occupying three quadrants, excluding the high stability change, high centrality quadrant (Figure 5d). Considering this shape, proteomic perturbation sensitivity is highest among the proteins bordering the central bend of the bivariate distribution, closest to the intersection of the four quadrants. However, the rank order of per‐protein proteomic perturbation sensitivity is challenging to determine from simple inspection.

We therefore developed a composite sensitivity score (CSS) to summarize the blended contribution of oxidation‐induced destabilization and eigenvector centrality on a per‐protein basis (Figure 5e). The CSS holistically predicts the degree of impact that adding or removing an oxidative PTM will yield on perturbing the joint cartilage proteome, where a higher score implies greater perturbation. Per‐community CSS sums reveal that metabolic stress response (sum 18.97, 46% of all studied proteins) as a community carries significant weight compared to contractile‐like (sum 10.80, 26% of all studied proteins), collagen and ECM biosynthesis (sum 5.73, 14% of all studied proteins), and phospholipase A2 inhibition (sum 5.67, 14% of all studied proteins). Examining just the CSS upper 50th percentile, we find that this distinction is magnified, where 60% (12 of 20) of these high‐impact proteins are drawn from the metabolic stress response community (Table 1). Interestingly, we detected oxidative PTM changes on the majority (15 of 20, 75%) of proteins comprising the CSS upper 50th percentile, with more than half (8 of those 15, 53%) detected as oxidative PTM loss in the old senolytic‐treated mouse (Figure 5f). This result highlights that senolytic‐driven reduction of oxidative PTM in old mice is disproportionally focused among the proteins most likely to impact the joint protein milieu. Examining the proteins of the top 10 CSS reinforces the endoplasmic reticular glycoprotein chaperones calreticulin and calnexin as well as their potential glycoprotein substrates fibrillin and aggrecan as key nodes in the joint proteome. We also note the 60S ribosomal protein L13a and 14‐3‐3ζ as intriguing proteins belonging to the top 10 CSS. L13a plays an important role in the heterotrimeric GAIT complex, which upon phosphorylation, promotes inflammation‐induced translational suppression in mouse macrophages (Arif et al., 2012). 14‐3‐3ζ has likewise been implicated in inflammatory responses (Munier et al., 2021). However, also salient is that 14‐3‐3ζ, which itself has a degree of chaperone activity, has been shown to promote cellular senescence when depleted—of particular import considering that we detect gain of oxidative PTM on 14‐3‐3ζ in both the young and old senolytic treatment conditions (Lee et al., 2014; Sluchanko et al., 2014; Xu et al., 2013).

**TABLE 1:** Proteins from this study of high composite sensitivity score, simultaneously the top 20 and top fiftieth percentile.

Gene name	Protein name	Uniprot ID	Louvain community	Old PTM sign	Young PTM sign	Composite sensitivity score
Calr	Calreticulin	P14211	Phospholipase A2 inhibition	−1	0	1.00
Rpl13a	60S ribosomal protein L13a	P19253	Metabolic stress response	0	0	0.94
Fbn1	Fibrillin−1	Q61554	Collagen and ECM biosynthesis	−1	−1	0.89
Ywhae	14‐3‐3 protein epsilon	P62259	Metabolic stress response	−1	0	0.89
Acan	Aggrecan core protein	Q61282	Collagen and ECM biosynthesis	−1	1	0.85
Canx	Calnexin	P35564	Metabolic stress response	−1	0	0.82
Hist1h4j	Histone H4	P62806	Metabolic stress response	−1	0	0.81
Tnnc2	Troponin C	P20801	Contractile‐like	0	0	0.78
H3f3a	Histone H3.3	P84244	Metabolic stress response	0	0	0.75
Ywhaz	14‐3‐3 protein zeta/delta	P63101	Metabolic stress response	1	1	0.71
Gapdh	Glyceraldehyde‐3‐phosphate dehydrogenase	A0A0A0MQF6	Phospholipase A2 inhibition	1	0	0.66
Rps8	40S ribosomal protein S8	P62242	Metabolic stress response	0	0	0.64
Hist2h3c2	Histone H3.2	P84228	Metabolic stress response	−1	0	0.61
Hist1h3g	Histone H3.1	P68433	Metabolic stress response	0	0	0.60
Hspa4	Heat shock 70 kDa protein 4	Q3U2G2	Metabolic stress response	0	0	0.60
Sptan1	Spectrin alpha chain	A3KGU7	Contractile‐like	0	0	0.55
Tpm1	Tropomyosin alpha‐1 chain	P58771	Contractile‐like	1	−1	0.55
Actb	Actin, cytoplasmic 1 (Beta‐actin)	P60710	Metabolic stress response	0	0	0.54
Rps11	40S ribosomal protein S11	P62281	Metabolic stress response	0	0	0.52
Actn4	Alpha‐actinin‐4	P57780	Contractile‐like	−1	0	0.51

*Note*: The PTM sign columns denote the net direction of the oxidative PTM change upon senolytic treatment in the indicated condition, where negative is a loss, zero is no change, and positive is a gain.

## 3 DISCUSSION

In this study, we interrogated the effect of senolytic treatment on knee oxidative stress after ACLT‐induced osteoarthritis, in both young and aged mice. The study employed in vivo timepoints for UBX0101 administration and terminal harvesting that were in alignment with previous work demonstrating diminished response to UBX0101 treatment in aged mice compared to their younger counterparts (Faust et al., 2020; Jeon et al., 2017). We observed active gene expression consistent with an oxidative stress response, as well as bulk protein oxidation levels which tracked with age. Senolytic treatment reduced knee protein oxidation in aged mice. These observations were corroborated by a bottom‐up MS analysis interrogating the OA joint proteome at the molecular level, detailing the senolytic‐driven loss of oxidation‐related PTMs in aged OA animals. Most strikingly, the MS analysis revealed opposing response patterns between young and old mouse senolytic treatments. A negative correlation relates young and old %Δ oxidative PTM per site. Young and old joint protein–protein interaction network eigenvector centrality correlate negatively and positively, respectively, as a function of %Δ PTM per site.

Our biophysically informed composite sensitivity score predicts that the proteins whose oxidative modification would be most influential in perturbing the joint cartilage proteomic milieu are also proteins where we experimentally detected modification, particularly oxidative PTM loss in old mice. We highlight that our composite scoring scheme nominates specific targets of high influence in the joint cartilage. We predict that perturbation of these targets, among the proteins studied here, are the most likely to modulate phenotypes related to age‐associated joint oxidation. Therefore, our high scoring targets represent a platform of justifiable candidates from which to initiate a hypothesis‐driven mechanistic study or therapeutic rational design effort.

While not mechanistically informative per se, the qualitative functional associations we draw from our analysis enable guided speculation about the differences between young and old senolytic treatment outcomes, as well as the biological inputs influencing those differences. Diverging patterns of protein abundance changes between young and old mice suggest that senolysis in young mice promotes a program of cartilage and chondrocyte development, consistent with prior work (Jeon et al., 2017). In contrast, old mice blend pathogenic‐associated cartilage and proteostatic stress upregulation, but dampen pathogenic‐associated immune signatures and ROS production. These categories roughly correspond with the four Louvain‐detected communities—cartilage biosynthesis, contractile‐like, phospholipase A2 inhibition, metabolic stress response—and roughly link protein level abundance changes to the protein–protein interaction network topology. The outlier category, a contractile‐like population, recalls an unexpected observation from a prior study wherein muscle‐related gene expression is detected in joint extracts explicitly devoid of muscle tissue (Loeser et al., 2012). While this may represent a joint cell population yet to be definitively identified, these might also correspond to contractile chondrocytes thought to arise in osteoarthritis (Kim & Spector, 2000; Lee et al., 2000; Povýsil et al., 2008). Alternatively, they may represent myofibroblast‐like cells, which have been previously observed in nearby post‐traumatic osteoarthritic synovium and infrapatellar fat pad (Knights et al., 2023; Song et al., 2016; Sono et al., 2020).

More broadly, the synthesis of our qualitative and quantitative insights hint at an altered homeostasis in the aged joint where cellular resource equilibria prioritize managing the proteostatic and oxidative stresses that naturally accumulate over the lifespan. In other words, our MS results raise a hypothesis wherein young and aged OA animal knee joint proteomes may be differently poised to respond to senolytic treatment. One consequence of this age‐dependent prioritization appears to include a failure to reestablish healthy cartilage matrix homeostasis, itself a characteristic feature of OA. Given this hypothesis, one might subsequently predict that after senolysis, aged OA animals would preferentially return to sustaining an entrenched proteomic stabilization phenotype instead of simply reverting to a youthful repair state.

Several facets of our data are consistent with this notion. First, aged animals uniquely responded to senolysis with upregulated cytosolic chaperone abundance, and downregulated ROS sources. Second, the majority of the most influential proteins of the joint proteome, associated with metabolic stress response, experience oxidative PTM loss in senolytic‐treated aged OA animals. These include endoplasmic reticular chaperones, where upon oxidative PTM loss, likely increase their chaperone activity with broad downstream effects on proteostatic health, especially on cartilage constituent glycoproteins. Third, senolytics drove broad reduction of oxidatively driven PTMs in aged OA animals, whether accounted for on a per‐modification or per‐protein basis, or measured in bulk, consistent with a homeostatic drive promoting protein oxidative integrity. Together, this data is consistent with a view that underlying senolytic response bias shifts over the lifespan from clearing a path for cartilage or chondrocyte regeneration instead to clearing a path for metabolic and proteostatic maintenance.

A prioritization of proteostasis might be recognized as a prioritization of a deeply conserved biochemical process underlying age‐associated biological decline, even outside the animal kingdom and the eukaryotic domain (Santra et al., 2019; Steiner, 2021). All living organisms contend with sustained accumulation of protein instability, in the form of protein misfolding, aggregation, and oxidative damage. Considering our results, it appears that the SASP, or the lack thereof, may indirectly modulate the proteostatic state of cells, underscoring an intimate integration of external signals broadcast by SnCs and a tissue's innate resiliency to oxidative insults. How the clearance of SnCs and their SASP mechanistically promotes reduction of oxidative PTMs remains to be determined.

One plausible circumstance is that after SnC clearance, remaining cells may degrade oxidatively modified proteins and replace them by synthesizing new, undamaged proteins. This might explain our observation of senolytic‐driven AGE and ALE loss, broadly thought irreversible (Vistoli et al., 2013). It is known that cells manage oxidative stress in part by depending on the proteasome's capability to degrade oxidized proteins, where the proteasome is a multi‐subunit enzyme central to the ubiquitin‐proteasome proteolytic pathway (Davies, 2001; Pickering et al., 2010). Interestingly, exposure to the SASP factor, IFN‐γ, has been shown to promote formation of an alternative proteasomal holoenzyme termed the immunoproteasome, involved in producing peptides for MHC class I display (Heink et al., 2005). If shifting the compositional equilibrium away from the constitutive proteasome and towards the immunoproteaseome alters a cell's capability to address accumulation of oxidative PTMs, then such a mechanism could represent one link between the tissue‐level paracrine SASP milieu and the intracellular states promoting proteomic health. Complementary immune‐related communication avenues may include 60S ribosomal protein L13a, a GAIT‐complex IFN‐γ responder protein, and 14–3‐3ζ, an inflammatory signaler directly regulating senescence, both of which our results highlight as influential in the aging joint proteome.

Such links, yet to be experimentally demonstrated, could open the possibility for senolytic treatments or anti‐inflammatory immunomodulatory treatments, such as neutralizing antibodies or biomaterial implants, to be leveraged as gateways to tune proteasomal activity either alone or in combination therapy (Faust et al., 2020; Sadtler et al., 2016). Future investigations should also examine the impact of sex differences, as the current study was conducted on male mice. Physiological sex disparities exist between male and female mice, which may impact their respective responses to injury, drug treatment, and age.

Ultimately, a therapeutic goal to address OA is to drive the reactivation of joint repair programs in aged tissue to restore normal cartilage homeostasis. Our observations reinforce the notion that removal of senescent cells and the correlative reduction of oxidative stress are insufficient to fully drive reversal of OA pathology in advanced age. The possibility remains that stabilizing proteomic health may be a necessary or concurrent step before initiating joint repair. Our study nominates sensitive targets of proteomic perturbation and oxidative rehabilitation in aged mice which may provide a foundation to investigate how cellular and tissue resources can be reallocated towards joint repair following senolysis. It also again emphasizes the biomedical needs for therapeutic development specifically targeting the unique attributes of an aged environment often requiring multiple targets, or combination therapy.

## 4 MATERIALS AND METHODS

### 4.1 Surgically induced OA mouse model and senolytic treatment

Anterior cruciate ligament transection (ACLT) surgeries were performed on 10 week or 19 month old male C57BL/6 mice from Charles River. The mice used for all experiments were randomly assigned to control or treatment groups and to those used in proteome analysis. For senolytic treatments, 6 doses of either vehicle control (5% DMSO and 95% phosphate buffer saline (PBS)) or 1 mM UBX0101 in 10 μL PBS solutions were intra‐articularly administered to the joint of the operated knee via a 30‐gauge needle. Injections were given every 2 days starting on day 14 after ACLT. Additional details are described in the Data S1.

### 4.2 Reverse transcription‐quantitative polymerase chain reaction (RT‐qPCR)

cDNA derived from homogenized mouse joint cartilage tissues were used as input for TaqMan Probe based qPCR. Details are described in the Data S1. The following Taqman probes (Applied Biosystems/ThermoFisher Scientific) were used. atf3: Mm00476033_m1, ddit4: Mm00512504_g1, mt1: Mm00496660_g1, and rer1 (housekeeping and inter‐plate calibrator target): Mm00471276_m1.

### 4.3 MS analysis of cartilage proteins from young and aged OA joints

Cartilage pieces harvested from young and aged mice OA joints 4 weeks after ACLT surgery with or without UBX0101 treatment were used to prepare biologically independent protein extracts derived from two mice for each sample set: young vehicle, young senolytic treated, old vehicle, old senolytic treated. Extracts were used as input to SDS‐PAGE and DNPH derivatization analysis, described in detail in the Data S1. Peptides eluted from in‐gel SDS‐PAGE LysC/trypsin/Glu‐C proteolytic digests were analyzed by nano liquid‐chromatography electrospray ionization tandem‐MS. For label‐free quantitation (LFQ) analysis, technical replicates (2 or 3 × 25 μg) from each digested cartilage protein preparation were analyzed on a Q Exactive HF quadrupole orbitrap mass spectrometer (Thermo Fisher Scientific) coupled to an Easy nLC 1000 UHPLC (Thermo Fisher Scientific) through a nanoelectrospray ion source. The mass spectrometer operation parameters and raw data files processing parameters are defined in the Data S1. The output was used in the protein identification, label‐free relative peptide quantification, and gene ontology enrichment analyses.

### 4.4 PTM stability analysis

Counts of positively (Arg, Lys), negatively (Asp, Glu), and variably (His) charged amino acids were summed on a per‐protein basis from reference proteomes described in the supplementary methods. Then, the folding free energy stability change was estimated according to methodology adapted from DeGraff et al., *Structure* (2016) (De Graff et al., 2016). The above counts were used to calculate protein net charges:

$$Q_{n}=\sum_{\text{residue}=1}^{N}C_{\text{positive}}+0.4*C_{\text{variable}}-C_{\text{negative}}$$

$$Q_{d}=\sum_{\text{residue}=1}^{N}C_{\text{positive}}+0.1*C_{\text{variable}}-C_{\text{negative}}$$

where the contribution of histidine differs between the native (*Q*_n_) and denatured (*Q*_d_) state and is accounted for via empirically estimated protonation fractions. Additional parameters taken to simulate cytoplasmic conditions included Boltzmann's constant, temperature (Kelvin), Bjerrum length *I*_b_ = 7.13 Å, and the inverse Debye screening length, *κ* = 0.03 Å^−1^. Protein native and denatured radii of gyration were estimated according to the Flory polymer scaling relationships *R*_n_ = 2.24*N ^0.392^ Å and *R*_d_ = 1.927*N ^0.598^ Å, respectively, as a function of the number of residues in the protein (*N*). Finally, the average change in a protein's folding free energy, ΔΔ*G*, resulting from addition or subtraction of a single charge was taken as:

$$\frac{\Delta \Delta G}{\mathrm{kT}}=\frac{I_{b}\left(\pm 2Q_{d}+1\right)}{2R_{d}\left(1+\kappa R_{d}\right)}-\frac{I_{b}\left(\pm 2Q_{n}+1\right)}{2R_{n}\left(1+\kappa R_{n}\right)}$$

### 4.5 Graph theory analysis and composite sensitivity score (CSS)

The *M. musculus* protein–protein interaction network topology was downloaded from the STRING database. Network centrality metrics were calculated across the full *M. musculus* proteome interaction network, then the proteins detected by mass spectrometry in this study were used to subset the network. The Louvain modularity‐optimizing community detection algorithm was applied to this subnetwork. Additional details are described in the supplementary methods.

The raw sensitivity score (RSS) is the product of min–max normalized protein stability change (ΔΔ*G*) upon oxidative charge‐change and min–max normalized eigenvector centrality (*E*) of a given protein. *P* refers to the set of all proteins represented in this study. The CSS is the RSS normalized in a similar fashion across RSS values.

$$\begin{array}{c} \mathrm{RSS}=\frac{∆∆G-\min_{P}\left(∆∆G\right)}{\max_{P}\left(∆∆G\right)-\min_{P}\left(∆∆G\right)}*\frac{E-\min_{P}\left(E\right)}{\max_{P}\left(E\right)-\min_{P}\left(E\right)} \end{array}$$

$$\mathrm{CSS}=\frac{\mathrm{RSS}-\min_{\mathrm{RSS}}\left(\mathrm{RSS}\right)}{\max_{\mathrm{RSS}}\left(\mathrm{RSS}\right)-\min_{\mathrm{RSS}}\left(\mathrm{RSS}\right)}$$

## AUTHOR CONTRIBUTIONS

Alexander F. Chin drafted the initial manuscript, performed MS analysis, and conceived, designed, and performed proteomic, biophysical, and graph theoretic analyses. Jin Han, Hong Zhang, and Younghwan Choi performed animal surgeries. Alexander F. Chin, Jin Han, and Younghwan Choi performed qPCR and qPCR analyses. Cristina C. Clement performed DNPH analysis, MS data collection, and associated raw data processing. Ok Hee Jeon conceived, performed, and designed in vivo imaging experiments and animal surgeries. Jin Han, Hong Zhang, and Maria Browne performed histology. Jennifer H. Elisseeff, and Ok Hee Jeon provided conceptual, analytic, and supervisory support. All authors reviewed and edited the manuscript.

## CONFLICT OF INTEREST STATEMENT

JHE is an equity holder of Unity Biotechnology, is a founder and equity holder of Aegeria Soft Tissue (AST), and is a consultant to Tessara.

## Supporting information


Data S1.

## Data Availability

The mass spectrometry proteomics data that support the findings of this study are openly available at the ProteomeXchange Consortium via the PRIDE partner repository at http://doi.org/10.6019/PXD031782, dataset identifier PXD031782.
