# Supplementary material for: Senolytic treatment reduces oxidative protein stress in an aging male murine model of post‐traumatic osteoarthritis
Source: Aging Cell. 2023 Sep 25;22(11):e13979. doi: 10.1111/acel.13979 (PMC10652304; doi:10.1111/acel.13979)
Supplement: Supplementary file 1 — Data S1. [file ACEL-22-e13979-s001.pdf]

Supplementary Information for

SENOLYTIC TREATMENT REDUCES OXIDATIVE PROTEIN STRESS IN AN AGING MALE MURINE MODEL OF POST-TRAUMATIC OSTEOARTHRITIS

Alexander F. Chin[1,△], Jin Han[1,△], Cristina C. Clement[2], Younghwan Choi[1], Hong Zhang[1], Maria Browne[1], Ok Hee Jeon[1,3*], and Jennifer H. Elisseeff[1,4*]

[1]Translational Tissue Engineering Center, Wilmer Eye Institute and Department of Biomedical Engineering, Johns Hopkins University School of Medicine, Baltimore, Maryland, USA
[2]Department of Radiation Oncology, Englander Institute for Precision Medicine, Weill Cornell Medicine, New York, New York, USA
[3]Department of Biomedical Sciences, Korea University College of Medicine, Seoul, Republic of Korea
[4]Bloomberg-Kimmel Institute for Cancer Immunotherapy, Johns Hopkins University School of Medicine, Baltimore, Maryland, USA

△AFC and JH contributed equally to this work.

*Correspondence: Ok Hee Jeon (ojeon@korea.ac.kr) and Jennifer H. Elisseeff (jhe@jhu.edu)

SUPPLEMENTARY METHODS

Surgically induced OA mouse model

Anterior cruciate ligament transection (ACLT) surgeries were performed on 10 week or 19 month old male C57BL/6 mice from Charles River (Germantown, MD, USA). The mice used for all experiments were randomly assigned to control or treatment groups and to those used in proteome analysis. Mice were placed under general anesthesia with 3% isoflurane, and hind limbs were shaved and prepared for aseptic surgery. The knee joint was exposed following a medial capsular incision and the ACL were transected with micro-scissors under a surgical microscope. After irrigation with saline to remove tissue debris, the skin incision was closed. All animal procedures were approved by the Institutional Animal Care and Use Committee at Johns Hopkins University School of Medicine.

Histology

Mouse knees were fixed in 4% paraformaldehyde, decalcified in 10% EDTA, then dehydrated and embedded in paraffin. For histological staining, sections 7µm thick were prepared and stained with Safranin-O / Fast Green. Images were captured with a Zeiss Axioimager A2 upright microscope using a Zeiss Plan-Apochromat 20x/0,8 objective.

Reverse transcription-quantitative polymerase chain reaction (RT-qPCR)

Mouse joint cartilage tissues were harvested then placed in 1mL Trizol reagent (ThermoFisher 15596026) and homogenized for 3 cycles of 15 seconds in an Omni BeadRuptor-12 bead mill homogenizer. After incubation on ice for five minutes, 200uL chloroform was added to the extract, vortexed for 30 seconds, then spun at maximum speed on a tabletop microcentrifuge for 15 minutes. The upper aqueous phase containing nucleic acid was reserved, then mixed with one volume of 70% EtOH. The resulting solution was applied to a microcentrifuge-column-based RNEasy Micro Kit (Qiagen 74034) for RNA extraction. The resulting RNA was checked for purity and concentration on a Nanodrop spectrophotometer, then immediately frozen at -80°C. A 2500ng RNA aliquot was recovered at room temperature and reverse transcribed using SuperScript IV VILO Master Mix (Invitrogen 11756050). 125ng of the resulting cDNA was used as input per qPCR reaction, each consisting of TaqMan Gene Expression Master Mix (Applied Biosystems 4370074) and a reaction-specific TaqMan Gene Expression Assay (FAM) (Applied Biosystems 4331182). The reactions were run in MicroAmp Optical 96-Well Reaction Plates (Applied Biosystems N8010560), and run on a StepOnePlus Real-Time PCR System (Applied Biosystems 4376600) in Quantitative Comparative Ct (ΔΔCt) mode for 40 cycles. ThermoFisher Connect Relative Quantification qPCR software was used to perform inter-plate calibration.

The following Taqman probes (Appled Biosystems / ThermoFisher Scientific) were used for RT-qPCR (atf3: Mm00476033_m1, ddit4: Mm00512504_g1, mt1: Mm00496660_g1, and rer1: Mm00471276_m1). Rer1 was used as a housekeeping target transcript and inter-plate calibrator target transcript. The remaining targets were chosen for their recognized association with oxidative stress (Han *et al.*, 2008).

RT-qPCR statistical analysis

All analyses of RT-qPCR data used the Livak method, where ΔΔCt values were calculated and reported as relative quantification values calculated by 2-ΔΔCt. Data are displayed as mean ±

s.d. Statistical analysis was performed using an unpaired t-test or one-way ANOVA with Tukey's corrections applied using GraphPad Prism v8, with statistical significance designated at $p \leq 0.05$. All groups were compared to each other with multiple comparison correction unless otherwise stated.

Protein extraction from cartilage from young and aged OA joints

To access synovial fluid at the articular joint, sacrificed animals had their hind limbs shaved, their skin removed, and their knee joints then exposed following a medial capsular incision under a stereomicroscope (Olympus, Tokyo, Japan). 10 uL of saline was injected several times into the open joint space. To remove the supernatant containing a protein clot, fibers, and particulate matter, the synovial fluid was centrifuged at 3000 rpm for 15 min and stored at -80°C until analysis. Cartilage pieces (extracted from young and aged mice OA joints 4 wk after ACLT surgery with or without UBX0101 treatment) were subjected to protein extraction procedures using a modified method previously developed for analysis of articular cartilage (Supplementary Figure 1) (Scharf *et al.*, 2013). Cartilage pieces from young and aged mice OA joints 4 wk after ACLT surgery with or without UBX0101 treatment first were equilibrated in 200 µl of sterile PBS for 15 min at 37 °C. The samples were subjected to digestion with 0.1 % hyaluronidase (3 units per sample) in PBS for 30 min at 37 °C to remove surface hyaluronate, followed by centrifugation at 14,000 rpm for 30 sec to remove the digested hyaluronate. The remaining pieces of cartilage were resuspended in 200 µl sterile PBS and treated with collagenase using 8 units of bacterial collagenase type II (Worthington Biochemical Corporation, Lakewood, NJ, USA) overnight at 37 °C. Protease inhibitor cocktail was then added to the total protein solution from each cartilage sample. Biologically independent protein preparations derived from two mice were performed for each sample set: young vehicle, young senolytic treated, old vehicle, old senolytic treated. Initial protein preparations were then subsequently processed with NaCl and guanidine hydrochloride extraction. Total protein solutions were subjected to 9 freeze/thaw cycles in alternating liquid nitrogen and 37 °C water baths. The sample solution conditions were adjusted to a salt buffer for protein extraction, 1M final NaCl in 100 mM Tris/acetate pH 8.0 and incubated for 18 hr at 4 °C on a shaker. After NaCl extraction, the samples were spun at 14,000 rpm for 30 min, the supernatant collected, and labeled E1 (extraction E1, corresponding to the NaCl extraction). The pellet from E1 extraction (consisting of proteins not soluble in 1M NaCl) was resuspended in 100 ul 4 M guanidine HCL, 65 mM dithiothreitol (DTT), 10 mM EDTA in 50 mM sodium acetate, pH 5.8 for 18 hr at 4 °C on a shaker. The insoluble guanidine hydrochloride (GdnHCl) treated fractions (extraction E2) were further spun at 14,000 rpm for 30 min in the microcentrifuge and the supernatant was collected and labeled E2 (protein extracted with GdnHCl). The GdnHCl extraction procedure was repeated one additional time for the pellet and the second supernatant was combined with the first E2 supernatant representing the total E2 extracted proteins with GdnHCl. The level of bone protein contamination from the cartilage extracts resulting from this procedure was estimated to be low (< 5%) (Supplementary figure 4).

SDS-PAGE analysis of cartilage proteins from young and aged OA joints

Protein preparations from both the NaCl soluble (E1) and GdnHCl soluble (E2) fractions were precipitated using 5 volumes of cold acetone (O/N, at −20 °C). The resulting pellets from acetone precipitation were collected by centrifugation at 14,000 rpm for 30 min in a microcentrifuge, and gently washed 3 times with 50 % acetone. The final pellets were resuspended in 100 µL solubilization buffer (7 M Urea, 2 M Thiourea, 4 % CHAPS in 30 mM

Tris, pH 8.0). Aliquots from the solubilized E1 and E2 extractions were used to determine the total protein concentration using a bicinchoninic acid assay (Thermo Scientific, IL, USA). Solubilized proteins processed in this manner were subjected to two separate SDS-PAGE procedures. In the first procedure, approximately 30 µg each of NaCl (E1) and GdnHCl (E2) extracted protein fractions from young and old OA joints from mice subjected to ACLT surgery, with or without UBX0101 treatment, were run on 1D SDS-PAGE pre-casted 4 % - 20 % gradient gels (Biorad). The gels were stained using a silver-staining kit (Thermo Scientific, IL, USA). Each sample lane was divided into 12 equal bands then cut out with a razor. The resulting gel pieces were placed in subjected to in-gel digestion by a combination of LysC/trypsin/Glu-C enzymes in 25 mM ammonium bicarbonate buffer, carried out at 37 °C overnight. In the second, separate procedure, protein bulk carbonylation was probed when aliquots of the E1 and E2 protein extracts were treated with 2,4-dinitrophenylhydrazone (DNPH) for 15 min at room temperature, neutralized, then run on a 1D SDS-PAGE pre-casted 4 % – 20 % gradient gel. Gel proteins were Western-transferred to a PVDF membrane, stained with rabbit anti-DNP primary antibody, and detected with goat anti-rabbit HRP-conjugated secondary antibody (Abcam, ab178020). Densitometry measurements were divided by known total protein per well as determined by the BCA assay, resulting in a normalized count of carbonylated protein. The resulting calculated degree of carbonylation reflects the overall quantity of protein oxidative stress products (Dalle-Donne *et al.*, 2003).

Mass Spectrometric protein identification and label-free relative peptide quantification (LFQ analysis)

The Q Exactive HF quadrupole orbitrap mass spectrometer (Thermo Fisher Scientific, Waltham, MA, USA) coupled to an Easy nLC 1000 UHPLC (Thermo Fisher Scientific, USA) through a nanoelectrospray ion source was operated in the positive ion mode, in the data–dependent acquisition (DDA) mode. Full MS scans were obtained with a range of m/z 300 to 1600 and a mass resolution of 120,000 at m/z 200. HCD collision was performed on the 15 most significant peaks, and tandem mass spectra were acquired at a mass resolution of 30,000 at m/z 200 and a target value of $1.00e^{+05}$ with a maximum injection time of 100 ms. Raw files from each technical and biological replicate were filtered, de novo sequenced and assigned with protein ID using Peaks 7.0, 7.5 and 8.0 software (Bioinformatics Solutions, Waterloo, Canada), by searching against the mouse (Mus musculus) Swiss-Prot database (82, 628 entries). The following search parameters were applied for LFQ analysis: trypsin, Lys-C and GluC restriction for enzymes and one allowed missed cleaved at one peptide end. The parent mass tolerance was set to 15-18 ppm using monoisotopic mass, and fragment ion mass tolerance was set to 0.05 Da. Carbamidomethyl cysteine (+57.0215 on C) was specified in PEAKS as a fixed modification. Methionine, lysine, proline, arginine, cysteine and asparagine oxidations (+15.99 on CKMNPR), deamidation of asparagine and glutamine (NQ-0.98) and pyro-Glu from glutamine (Q-18.01 N-term) were set as variable modifications. Data were validated using the FDR method built in the PEAKS 7.0-8.0 and protein identifications were accepted if they could be assigned with a confidence score (-10logP)>15 for peptides and (-10logP)>15 for proteins; a minimum of 1 peptide per protein after data were filtered for less than 0.8% FDR for peptides and less than 1% FDR for proteins identifications ($p<0.05$). An independent validation of the MS/MS-based peptides and protein identification was performed with Scaffold (version Scaffold_4.6.2, Proteome Software Inc.) using the compatible ".mzid" files of all cartilage samples exported from PEAKS 7.0-8.0. The Scaffold built in option "MuDPIT" was used to combine multiple files from biological and/or

technical replicates of each cartilage sample group: vehicle treated (Veh) and senolytic treatment (UBX0101). Peptide identifications were accepted if they could be established at greater than 95.0% probability by the Peptide Prophet algorithm with Scaffold delta-mass correction. Protein identifications were accepted if they could be assigned at greater than 90.0% probability and contained at least 1 identified peptide. Proteins that contained similar peptides and could not be differentiated based on MS/MS analysis alone were grouped to satisfy the principles of parsimony. LFQ was performed using the PEAKS Quantitative "Q" module (Bioinformatics Solution Inc., version 7.5-8.0). The data were filtered, smoothed, and aligned in retention time, followed by feature and precursor ion quantification detection based on peak volume and isotopic clustering using the built-in algorithm. Relative protein abundance was assigned to representative proteins of each protein group, by unique name or parent accession number, using MS2 total unique peptide counts. To determine differences in abundance between two given conditions, Fisher's exact test was used with a $p<0.05$ ($-\log10(p) = 1.30$) threshold to classify significant change. To determine changes in PTMs, site-specific peptide spectral counts were summed on a per-protein basis. Sums from biologically independent replicates were combined. Then, the percentage of sites post-translationally modified was calculated from the proportion of modified spectral counts divided by the total spectral counts. Changes in PTM between experimental conditions were calculated as the difference in the PTM percent between conditions, either on a per-protein or a total per-modification basis. Between certain conditions, spectral counts meeting the detection confidence criteria outlined above were present in one condition and absent in the other, in some cases precluding calculation of the PTM percent change for a specific protein or PTM, e.g., due to a division by zero operation. When described in the text or figure legends, the act of dropping data with unpaired spectral counts refers to the elimination of these specific protein or PTM entries in the displayed analysis. Gene ontology enrichment analysis was performed using the PANTHER classification system web interface at http://geneontology.org/ or http://pantherdb.org/ using default statistical settings (only considering enriched hits FDR $p < 0.05$). The mass spectrometry proteomics data that support the findings of this study are openly available at the ProteomeXchange Consortium via the PRIDE partner repository (Perez-Riverol *et al.*, 2022) at http://doi.org/10.6019/PXD031782, dataset identifier PXD031782.

Reference proteomes and PTM stability analysis

Reference proteomes for human (H. sapiens, UP000005640), mouse (M. musculus, UP000000589), Naked mole-rat (H. glaber, UP000006813), and Brandt's Bat (M. brandtii, UP000052978) were acquired from the Universal Protein Resource (UniProt) database (https://www.uniprot.org/). For each protein sequence, an initial sequence validation filtering step was applied in which selenocysteines were substituted with cysteines and all other non-standard amino acids were substituted with alanine. The resulting filtered proteomes were used as input to the PTM stability analysis. The in turn resulting stability change values, $\Delta\Delta G$, were used to annotate 2-dimensional kernel density estimates relating the absolute value of net charge against protein length across the above reference proteomes, and map proteins with detected post-translational modifications onto those empirical density estimates.

Graph theory analysis

The *M. musculus* protein-protein interaction network topology and node aliases were downloaded from the STRING database (Taxonomy ID 10090, database version 11.5) then parsed with NetworkX as an undirected graph (Szklarczyk *et al.*, 2019; Hagberg *et al.*, 2008). Of the 243 unique proteins represented in the mass spectrometry PTM analysis, 238 (98%) were successfully mapped to the interaction network via common Ensembl EntrezGene IDs, then used for subsequent graph theory analysis. Network centrality metrics were calculated across the 21,319 proteins and 7,248,180 edges represented in the full *M. musculus* proteome available from STRING and resultant measures were assigned to individual nodes, where eigenvector centralities are computed via the principal (all positive entries) eigenvector of the graph adjacency matrix (Bonacich, 1971). After assignment, the 238 proteins in this study were used to subset the network, without modifying the numerical centrality values associated with each node. The Louvain modularity-optimizing community detection algorithm was applied to this subnetwork (Blondel *et al.*, 2008). The resulting communities were examined by Reactome pathway and Gene Ontology analysis and described according to overrepresented terms and supporting literature (Gillespie *et al.*, 2022; Consortium, 2019; Bomalaski & Clark, 1993; Pruzanski *et al.*, 1985; Dore & Boilard, 2019; Kim *et al.*, 2009; Griveau *et al.*, 2018). Analyses were visualized with UpSet, Gephi, and Seaborn (Lex *et al.*, 2014; Bastian *et al.*, 2009; Waskom, 2021).

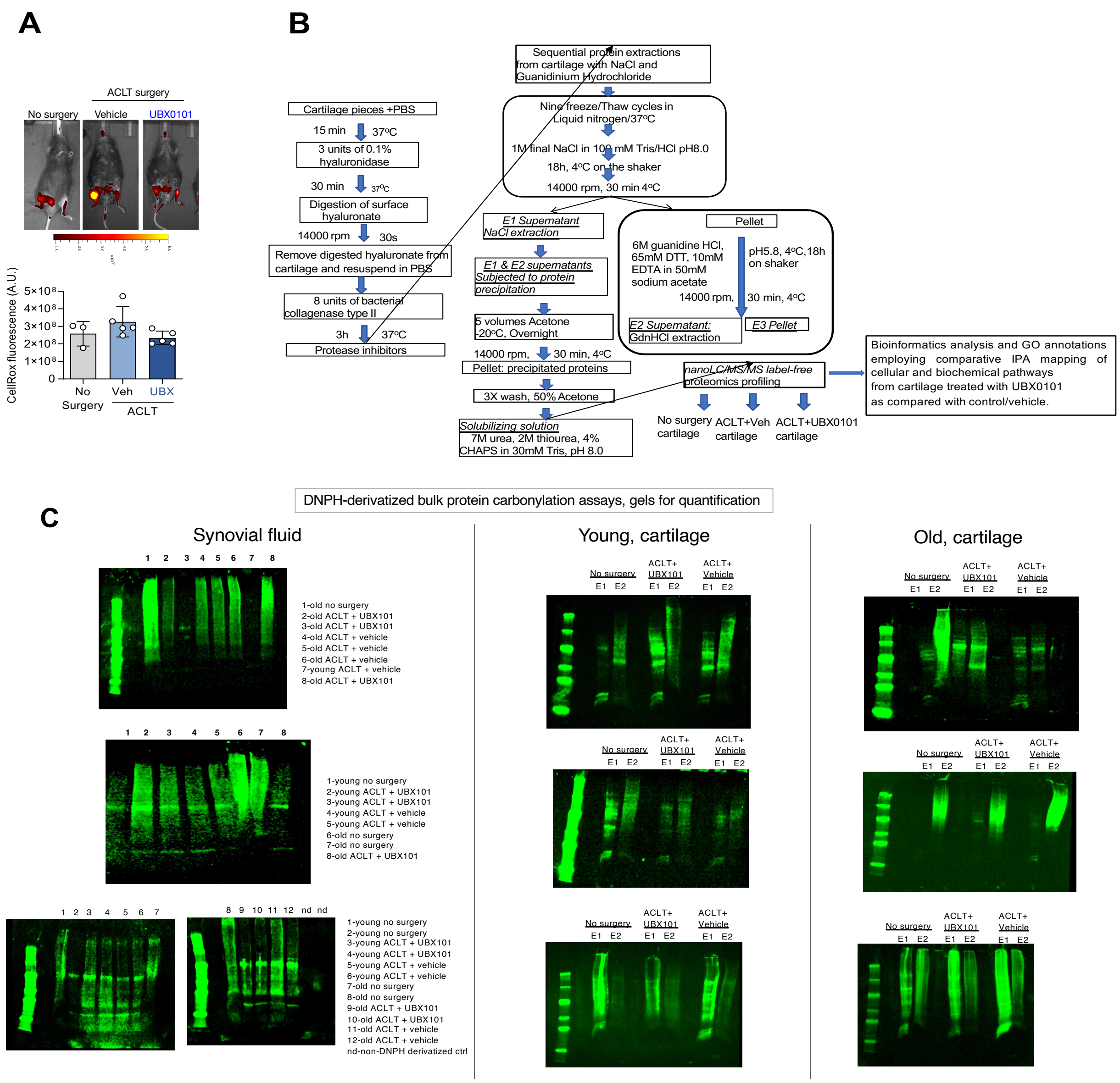

Supplementary Figure 1. (A) Images (top) and quantification (bottom) of CellROX fluorescence in old mice after injury or treatment. Mice were pre-treated with CellROX Deep Red, a fluorogenic probe used to measure cellular oxidative stress. Color bar scale represents calibrated radiant efficiency, in units of (p/sec/cm2/sr)/(µW/cm2). n=3 for No Surgery, n=5 for Veh and UBX. **(B)** schematic of the protein extraction process from mouse articular joint cartilage, depicted here feeding into MS analysis. **(C)** Carbonylated protein extracts were derivatized with 2,4-dinitrophenylhydrazone, forming a dinitrophenyl (DNP) hydrazone product, which in turn was probed in a Western Blot by an anti-DNP antibody. Shown are representative scans of anti-DNPH probed, DNPH-derivatized joint-protein extract, used to estimate joint protein carbonylation.

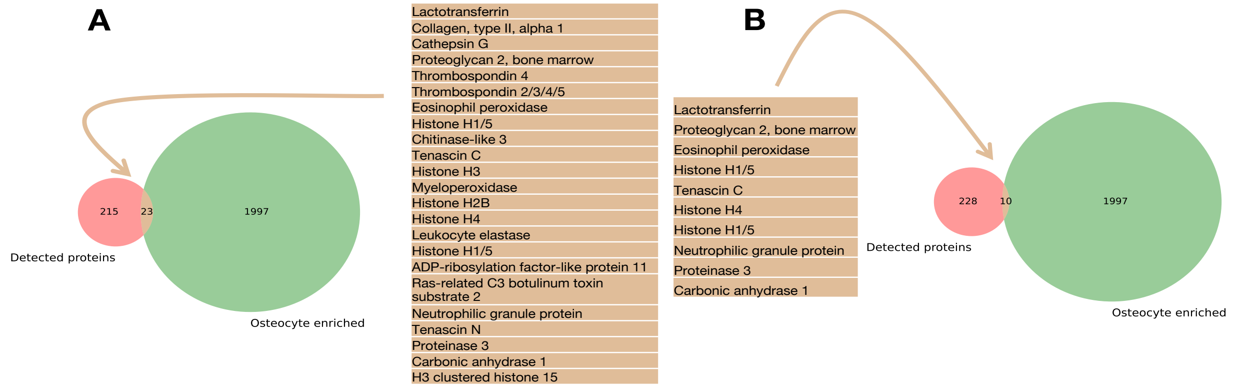

Supplementary Figure 2. Estimated proportion of bone-derived contamination of cartilage proteins in this study is low. (A) The count and identity of transcripts predicted to have high osteocyte specificity (defined as Tau > 0.85, annotated by (Youlten *et al.*, 2021)) were compared to the count and identity of proteins detected by mass spectrometry in this study which could be successfully aliased by Ensemble EntrezGene alias to the osteocyte specific transcripts (238 aliased / 243 proteins = 98% success rate). Of the proteins represented in the intersection of these sets, three highly expressed and seven modestly expressed are experimentally annotated as not bone specific (Three highly expressed: Cathepsin G (Ctsg), leukocyte elastase (Elane), myeloperoxidase (Mpo). Seven modestly expressed: Chitinase-like 3 (Chil3), Histone H3 (H3c11), Histone H2B (Hist1h2bh), ADP-ribosylation factor-like protein 11 (Arl11), Ras-related C3 botulinumtoxin subtrate 2 (Rac2), Tenascin N (Tnn) and H3 clustered histone 15 (H3c15)) (Uhlén *et al.*, 2015). Furthermore, three proteins are experimentally detected in cartilage or chondrocytes: Thrombospondin 2/3/4/5 (Comp / cartilage oligomeric matrix protein), thrombospondin 4 (Thbs4), and collagen type 2 alpha 1 (Col2a1). (B) After accounting for these thirteen non-osteocyte-specific transcripts, 4.8% (10 intersection / 238 detected) of the proteins in this study remain potentially derived from bone extract.

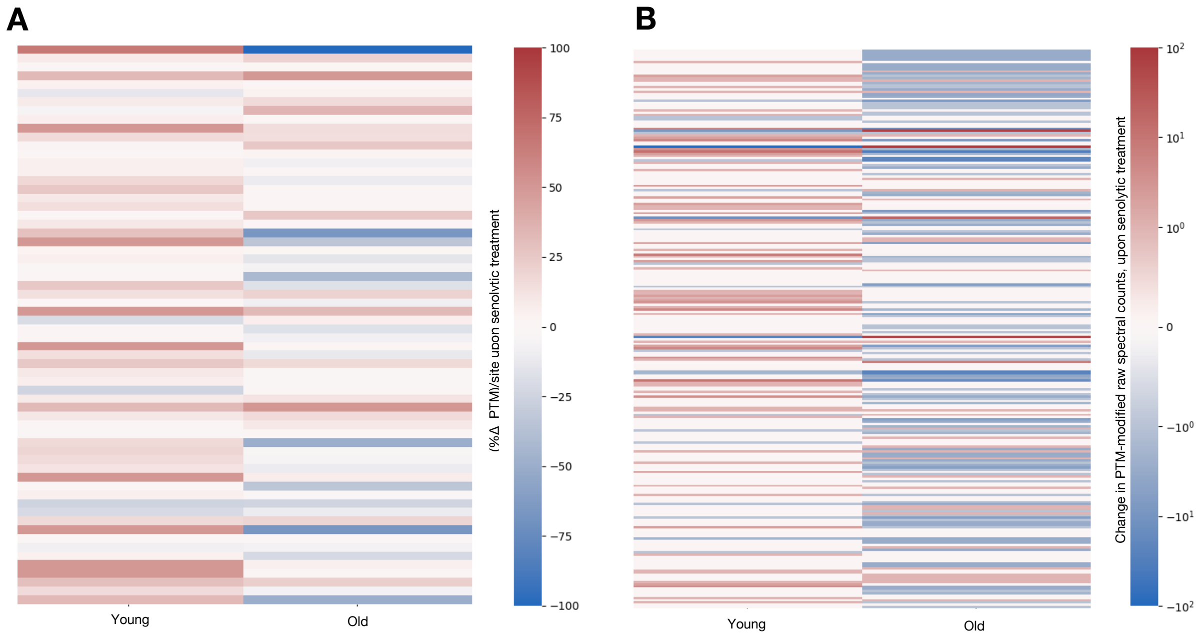

Supplementary Figure 3. (A) Heatmap representation of joint PTM changes in young and old OA mice, grouped by protein. This heatmap is an alternative representation of Figure 2B, except with the strict conditional requirement that percent change PTM per site for any given protein is both present and yields a real number result in both the young and aged mouse samples. 65 unique proteins are represented. (B) Heatmap representation of joint PTM changes in young and old OA mice, grouped by protein. This heatmap is an alternative representation of Figure 2B, except with the color scale reflecting changes in PTM-modified raw spectral counts. 243 unique proteins are represented.

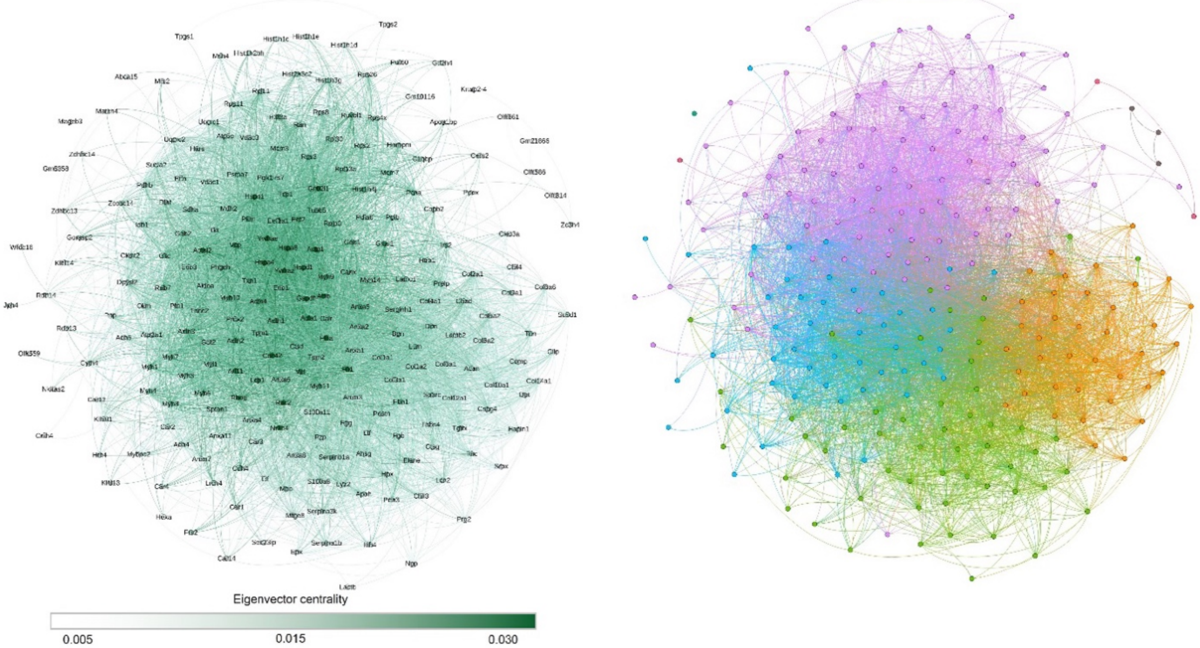

Supplementary Figure 4. Network graph illustrations of the OA joint proteins in this study (238 proteins and 6050 interactions), equivalent to those appearing in Figure 4, except (**Left)** with the network nodes colored by eigenvector centrality or, **(Right)** with network nodes alternatively unlabeled with common protein names.

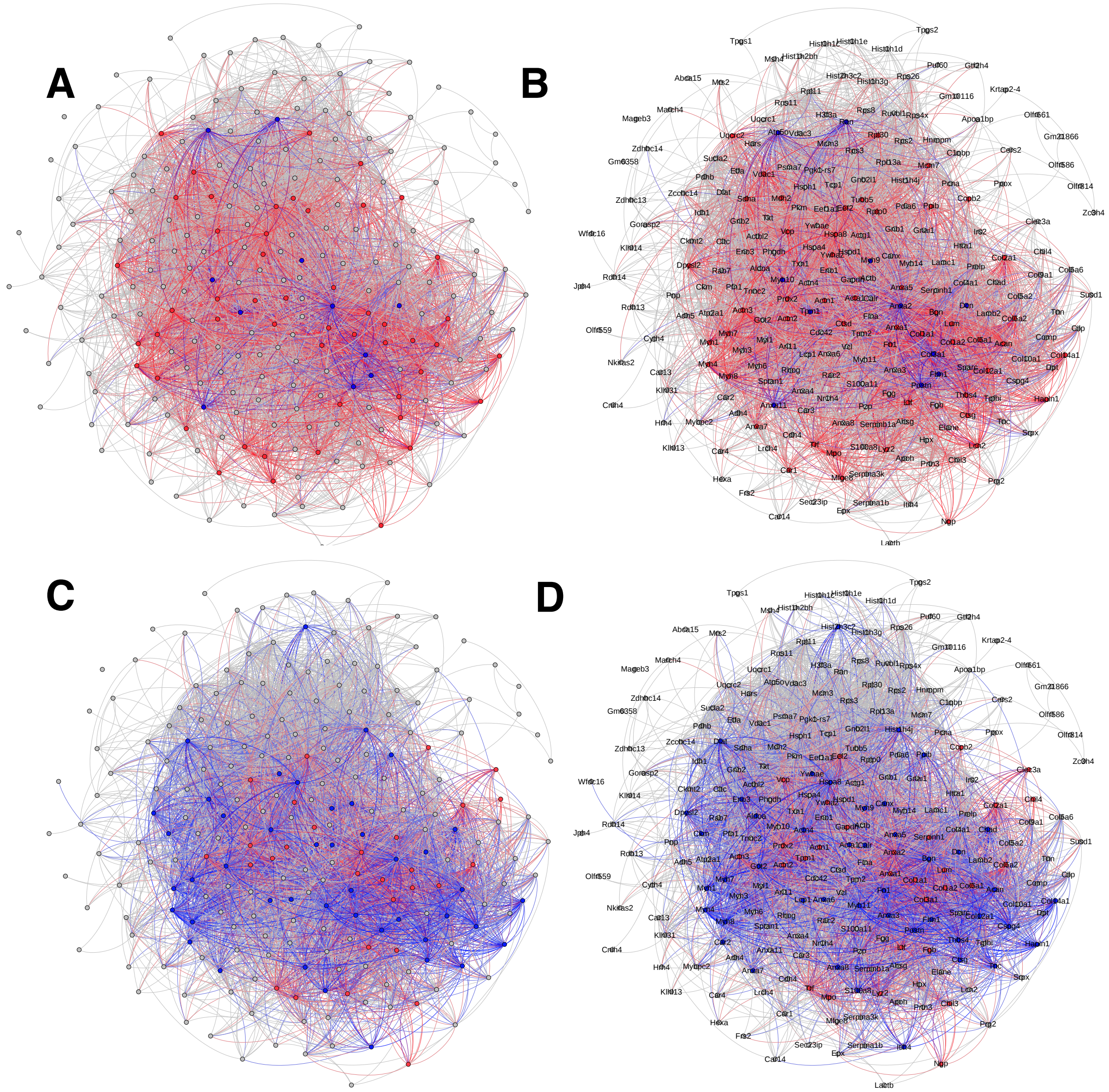

Supplementary Figure 5. Network graph illustrations of the OA joint proteins in this study (238 proteins and 6050 interactions), equivalent to those appearing in Figure 4, except with nodes and associated edges colored according to whether %Δ PTM > 0 (red) or %Δ PTM < 0 (blue). %Δ PTM = 0 are colored gray. (A) Senolytic-treated young mice, without or (B) with gene names. (C) Senolytic-treated old mice, without or (D) with gene names.

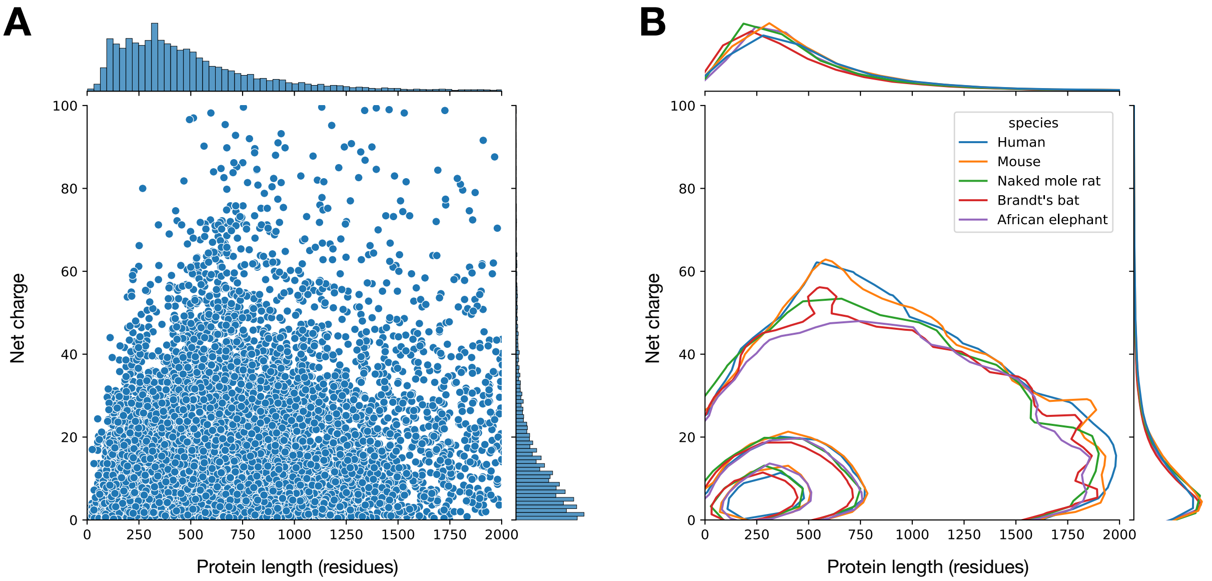

Supplementary Figure 6. Long lived mammals are equipped with proteomes biased toward lesser protein net charge and length. (A) The human proteome mapped on to the oxidative susceptibility space, one point per protein (20,600 proteins). (B) Complete proteome data mapped on to the oxidative susceptibility space but summarized with lines representing levels of a kernel density estimate of that data. Each line separates regions accounting for 25% of the proteins in that proteome. The naked mole rat (H. glaber), Brandt's bat (M. brandtii) and African elephant (L. africana) were selected as mammals with well-established unusually long lifespans.

Protein Accession	Protein Name	Young : %Δ PTM/site	Aged : %Δ PTM/site	Comments
O08553|DPYL2	Dihydropyrimidinase-related protein 2	66.7	-100.0	
P24369|PPIB	Peptidyl-prolyl cis-trans isomerase B	28.6	-66.7	
Q80X19|COEA1	Collagen alpha-1(XIV) chain	50.0	-66.7	
Q07076|ANXA7	Annexin A7	16.7	-50.0	
Q9Z1T2|TSP4	Thrombospondin-4	33.3	-50.0	Presence associated with OA (Maly *et al.*, 2019)
P29391|FRIL1	Ferritin light chain 1	0.0	-42.9	
P28293|CATG	Cathepsin G	50.0	-33.3	Protease for the joint lubricator protein lubricin (Huang *et al.*, 2020)
Q61233|PLSL	Plastin-2	0.0	-33.3	Required for osteoblast differentiation (Wang *et al.*, 2018)
Q61554|FBN1	Fibrillin-1	-25.6	-25.0	
Q91Z83|MYH7	Myosin-7	5.0	-21.5	
P48036|ANXA5	Annexin A5	25.0	-16.7	Required for osteoblast differentiation (Genetos *et al.*, 2014)
P62259|1433E	14-3-3 protein epsilon	0.0	-16.7	
P28653|PGS1	Biglycan	5.8	-14.3	Presence associated with OA (Barreto *et al.*, 2015)
P63017|HSP7C	Heat shock cognate 71 kDa protein	14.3	-12.5	
Q62009|POSTN	Periostin	-20.8	-10.4	

Table S1. Top fifteen decreases in percent PTM per site change upon senolytic treatment of aged OA mice. Some GO terms are over-represented in this set, as follows. GO molecular function: “ECM structural constituent,” “glycosaminoglycan binding.” GO cellular component: “myosin filament”, “collagen-containing ECM”, “axon terminus” and “cytoskeleton”. GO biological process: “movement of cell or subcellular component”.

**Upregulated**						
**Accession**	**Protein Name**	**Log2(Fold Change)**	**-Log10(p-value)**	**Aged : %Δ PTM/site**	**Young : %Δ PTM/site**	**Comments**
O88322|NID2	Nidogen-2	3.700	1.963			Basement membrane protein. Increase associated with OA (Krügel *et al.*, 2008).
Q9JM99|PRG4	Proteoglycan 4	3.459	1.601			Also known as lubricin, lubricates the synovial fluid and articular cartilage surface.
O35643|AP1B1	AP-1 complex subunit beta-1	3.322	1.423			
Q9DCD0|6PGD	6-phosphogluconate dehydrogenase, decarboxylating	3.322	1.423			
O35639|ANXA3	Annexin A3	2.807	1.611	-50.0		
O08553|DPYL2	Dihydropyrimidinase-related protein 2	2.415	1.506	-100.0	66.7	
P16110|LEG3	Galectin-3	2.415	1.506			Basement membrane protein. Reinforces cartilage lubrication via lubricin. Increase associated with OA (Janelle-Montcalm *et al.*, 2007; Reesink *et al.*, 2016).
Q7TPR4|ACTN1	Alpha-actinin-1	2	1.792	19.3	16.7	
P26443|DHE3	Glutamate dehydrogenase 1, mitochondrial	1.893	1.551			
Q9R118|HTRA1	Serine protease HTRA1	1.874	1.349			
Q68FD5|CLH1	Clathrin heavy chain 1	1.848	1.910			
Q64478|H2B1H	Histone H2B type 1-H	1.692	1.832			
P60710|ACTB	Actin, cytoplasmic 1	1.585	2.353			
O35744|CHIL3	Chitinase-like protein 3	1.493	2.224	1.8	0.0	
Q62009|POSTN	Periostin	1.466	1.745	-10.4	-20.8	Cartilage extracellular matrix protein
**Downregulated**						
**Accession**	**Protein Name**	**Log2(Fold Change)**	**-Log10(p-value)**	**Aged : %Δ PTM/site**	**Young : %Δ PTM/site**	**Comments**
P29699|FETUA	Alpha-2-HS-glycoprotein	-0.737	1.353	0.0		Also known as fetuin-A. Decreases linked with OA disease progression (Ozkan *et al.*, 2020).
Q5XKE0|MYPC2	Myosin-binding protein C, fast-type	-1.044	4.417			
P58771|TPM1	Tropomyosin alpha-1 chain	-1.070	3.048	6.7	-20.0	
Q5SX40|MYH1	Myosin-1	-1.078	5.120	-5.1	16.1	
P05977|MYL1	Myosin light chain 1/3, skeletal muscle isoform	-1.126	3.537	0.0		
P11087|CO1A1	Collagen alpha-1(I) chain	-1.155	15.439	25.7	2.7	Increased deposition associated with late-stage OA (Miosge *et al.*, 2004).
Q00897|A1AT4	Alpha-1-antitrypsin 1-4	-1.170	1.613			
Q8VHX6|FLNC	Filamin-C	-1.254	4.787			
P21550|ENOB	Beta-enolase	-1.441	3.449	-50.0		
O35367|KERA	Keratocan	-1.459	2.182			
O88990|ACTN3	Alpha-actinin-3	-1.648	8.654	0.46	4.76	
P49290|PERE	Eosinophil peroxidase	-1.808	1.705			
P20801|TNNC2	Troponin C, skeletal muscle	-2.170	2.365			
P58774|TPM2	Tropomyosin beta chain	-2.170	2.365	0.0		
Q62234|MYOM1	Myomesin-1	-3.248	5.969			

Table S2. Top fifteen changes in protein abundance upon senolytic treatment of young OA mice. Where available, PTM information is included as percent PTM per site change. Some GO terms are statistically over-represented in these sets, as follows. Upregulated GO cellular component: "collagen-containing ECM." Downregulated GO molecular function: "structural constituent of cytoskeleton" and "actin filament binding." Downregulated GO cellular component: "muscle thin filament tropomyosin" and "myosin filament." Downregulated GO biological process: "muscle contraction".

Upregulated						
**Accession**	**Protein Name**	**Log2(Fold Change)**	**-Log10(p-value)**	**Aged : %Δ PTM/site**	**Young : %Δ PTM/site**	**Comments**
Q91Z83|MYH7	Myosin-7	3.000	2.669	-21.474	5.000	
O08677|KNG1	Kininogen-1	2.807	2.285			Upregulated in OA diseased cartilage (Wu & Pasierb, 2011).
P08121|CO3A1	Collagen alpha-1(III) chain	2.778	13.811	35.637	-5.293	Concurrent deposition with collagen-II can indicate competing repair and OA pathology (Hosseininia *et al.*, 2016).
Q9EPW4|CLC3A	C-type lectin domain family 3 member A	2.585	1.908	33.333		Upregulated in OA diseased cartilage (Karlsson *et al.*, 2010).
P07309|TTHY	Transthyretin	2.322	1.538			Upregulated in OA diseased cartilage (Akasaki *et al.*, 2015).
O35701|MATN3	Matrilin-3	2.170	2.492			Cartilage extracellular matrix component.
Q00897|A1AT4	Alpha-1-antitrypsin 1-4	1.737	2.388			OA protective, reduces inflammation (Kaneva *et al.*, 2021)
Q80X90|FLNB	Filamin-B	1.737	2.388			Required for proper chrondrocytic activity in bone development (Lu *et al.*, 2007).
Q64478|H2B1H	Histone H2B type 1-H	1.678	6.574			
P09528|FRIH	Ferritin heavy chain	1.585	1.470			
P20152|VIME	Vimentin	1.585	6.483			
P97807|FUMH	Fumarate hydratase, mitochondrial	1.585	1.470			
Q8R422|CD109	CD109 antigen	1.222	1.445			
P23953|EST1C	Carboxylesterase 1C	1.138	1.996			
P28481|CO2A1	Collagen alpha-1(II) chain	1.070	3.294	2.155	3.684	
**Downregulated**						
**Accession**	**Protein Name**	**Log2(Fold Change)**	**-Log10(p-value)**	**Aged : %Δ PTM/site**	**Young : %Δ PTM/site**	**Comments**
P09103|PDIA1	Protein disulfide-isomerase	-1.708	1.843			
P14824|ANXA6	Annexin A6	-1.710	1.474	-33.333		Found in OA cartilage, required for IL-1 mediated cartilage damage, mediates NF-κB (Kirsch *et al.*, 2013; Jeon *et al.*, 2010).
P21956|MFGM	Lactadherin	-1.826	1.771	0	10	Exosomal component. Exosomes from senescent chondrocytes have been named biomarkers of OA (Jeon *et al.*, 2019).
P27005|S10A8	Protein S100-A8	-2.059	1.559	-12.281		
Q8VCM7|FIBG	Fibrinogen gamma chain	-2.290	2.923			
O35744|CHIL3	Chitinase-like protein 3	-2.293	11.454	1.824	0	
Q9QZZ6|DERM	Dermatopontin	-2.503	1.530			
Q9CZ13|QCR1	Cytochrome b-c1 complex subunit 1, mitochondrial	-2.700	1.353			
P97429|ANXA4	Annexin A4	-2.907	1.656			Modulates NF-κB immune pathway, which when overstimulated promotes OA(Rigoglou & Papavassiliou, 2013)
Q9DB77|QCR2	Cytochrome b-c1 complex subunit 2, mitochondrial	-2.907	1.656	0	50	
P15532|NDKA	Nucleoside diphosphate kinase A	-3.459	1.508			
P99024|TBB5	Tubulin beta-5 chain	-3.700	1.851		5	
P84228|H32	Histone H3.2	-4.170	2.738	-9.091		
Q66K08|CILP1	Cartilage intermediate layer protein 1	-4.585	3.836			Accumulates with age, stimulates OA-promoting immune response (Lorenzo *et al.*, 1998; Tsuruha *et al.*, 2001).
Q62351|TFR1	Transferrin receptor protein 1	-5.129	5.905			

Table S3. Top fifteen changes in protein abundance upon senolytic treatment of aged OA mice. Where available, PTM information is included as percent PTM per site change. Some GO terms are statistically over-represented in these sets, as follows. Upregulated GO cellular component: "fibrillar collagen trimer" and "extracellular space". Downregulated GO molecular function: "protein-containing complex binding". Downregulated GO cellular component: "mitochondrial respiratory chain complex III" and "collagen-containing ECM".

Young			
**Accession**	**Protein Name**	**Log2(Fold Change)**	**-Log10(p-value)**
P62821|RAB1A_MOUSE	Ras-related protein Rab-1A	-10.0	2.5
Q9D0F9|PGM1_MOUSE	Phosphoglucomutase-1	-10.0	2.9
GRP78_MOUSE	Endoplasmic reticulum chaperone BiP	-10.0	5.4
P07310|KCRM_MOUSE	Creatine kinase M-type	-10.0	4.6
Q5SX39|MYH4_MOUSE	Myosin-4	-1.3	20.0
O54724|PTRF_MOUSE	Caveolae-associated protein 1	10.0	1.5
O55023|IMPA1_MOUSE	Inositol monophosphatase 1	10.0	1.5
P20029|GRP78_MOUSE	Endoplasmic reticulum chaperone BiP	10.0	6.3
P17426|AP2A1_MOUSE	AP-2 complex subunit alpha-1	10.0	1.9
Q01320|TOP2A_MOUSE	DNA topoisomerase 2-alpha	10.0	1.5
P60122|RUVB1_MOUSE	RuvB-like 1	10.0	2.1
Q9JHU4|DYHC1_MOUSE	Cytoplasmic dynein 1 heavy chain 1	10.0	2.3
Q99KC8|VMA5A_MOUSE	von Willebrand factor A domain-containing protein 5A	10.0	2.1
Q11011|PSA_MOUSE	Puromycin-sensitive aminopeptidase	10.0	1.5
Q71LX4|TLN2_MOUSE	Talin-2	10.0	1.5
Q8C6K9|CO6A6_MOUSE	Collagen alpha-6(VI) chain	10.0	1.7
Q8CGP1|H2B1K_MOUSE	Histone H2B type 1-K	10.0	1.9
Q8VCH0|THIKB_MOUSE	3-ketoacyl-CoA thiolase B, peroxisomal	10.0	1.5
Q8VDN2|AT1A1_MOUSE	Sodium/potassium-transporting ATPase subunit alpha-1	10.0	1.5
**Aged**			
**Accession**	**Protein Name**	**Log2(Fold Change)**	**-Log10(p-value)**
P02463|CO4A1_MOUSE	Collagen alpha-1(IV) chain	-10.0	1.8
P22907|HEM3_MOUSE	Porphobilinogen deaminase	-10.0	1.4
P68368|TBA4A_MOUSE	Tubulin alpha-4A chain	-10.0	2.4
Q00898|A1AT5_MOUSE	Alpha-1-antitrypsin 1-5	-10.0	1.4
P11087|CO1A1_MOUSE	Collagen alpha-1(I) chain	2.7	20.0
P05213|TBA1B_MOUSE	Tubulin alpha-1B chain	10.0	2.2
CO5A2_MOUSE	Collagen alpha-2(V) chain	10.0	3.0
P07901|HS90A_MOUSE	Heat shock protein HSP 90-alpha	10.0	2.6
P68369|TBA1A_MOUSE	Tubulin alpha-1A chain	10.0	2.6
P06909|CFAH_MOUSE	Complement factor H	10.0	1.7
Q9JII6|AK1A1_MOUSE	Aldo-keto reductase family 1 member A1	10.0	2.6
P43277|H13_MOUSE	Histone H1.3	10.0	4.3
Q8CGP1|H2B1K_MOUSE	Histone H2B type 1-K	10.0	1.7
H2A1_MOUSE	Histone H2A type 1-B	10.0	4.7
P10922|H10_MOUSE	Histone H1.0	10.0	2.2

Table S4. Protein abundance change outliers. Some GO terms are statistically over-represented in these sets, as follows. Aged, downregulated GO molecular function: “platelet-derived growth factor binding”, “ECM structural constituent conferring tensile strength”, “structural constituent of cytoskeleton”. Aged, downregulated GO cellular component: “fibrillar collagen trimer” and “nucleosome”.

Louvain	Proteins	Reactome	GO biological process	GO molecular function	GO cellular component
Phospholipase A2 inhibition	Hexa, Hpx, Apoh, Fgg, Car2, Chil3, Anxa5, Hrh4, Car1, Gapdh, Chil4, Elane, Mfge8, Serpinb1a, Mpo, Anxa7, Fgb, Calr, Nr1h4, Itih4, Serpina1b, Anxa4, Lyz2, Irs2, S100a11, Ahsg, Prg2, Adh4, Car4, Lcn2, Serpina3k, Fn1, S100a8, Anxa1, Car14, Anxa11, Trf, Anxa8, Ltf, Ctsd, Lactb, Ngp, Pzp, Rdh13, Anxa3, Frs2, Prtn3, Car13, Anxa2, Got2, Car3, Ctsg, Epx	Neutrophil degranulation, Platelet degranulation, Response to elevated platelet cytosolic Ca2+, Innate Immune System, Antimicrobial peptides, Immune system, Metal sequestration by antimicrobial proteins, Platelet activation, signaling and aggregation, GRP2:SOS provides linkage to MAPK signaling for Integins, p130Cas linkage to MAPK signaling for integrins, Signaling by Interleukins	negative regulation of phospholipase A2 activity , negative regulation of phospholipase activity , negative regulation of hydrolase activity , regulation of hydrolase activity , regulation of catalytic activity , regulation of molecular function , biological regulation , negative regulation of catalytic activity , negative regulation of molecular function , defense response to nematode	phospholipase A2 inhibitor activity, phospholipase inhibitor activity, lipase inhibitor activity, enzyme inhibitor activity, enzyme regulator activity, molecular function regulator activity, N-acetyl-beta-D-galactosaminidase activity, carbonate dehydratase activity, hydro-lyase activity, carbon-oxygen lyase activity	fibrinogen complex, extracellular space, extracellular region, blood microparticle, azurophil granule, primary lysosome, lysosome, lytic vacuole, vacuole, cytoplasm
Contractile-like	Cltc, Myh9, Myh6, Lcp1, Cyth4, Klhl14, Ckm, Myh14, Abca15, Lrch4, Tnnc2, Actn1, Anxa6, Rab7, Acta1, Aldoa, Klhl31, Myl1, Myh1, Myh3, Myh10, Nkiras2, Flna, Rhog, Tpm1, Cdh4, Klhl13, Pfn1, Myh8, Actbl2, Wfdc16, Actn2, Rac2, Myh7, Mybpc2, Arl11, Sptan1, Atp2a1, Ckmt2, Actn4, Cdc42, Cnih4, Vcl, Actn3, Myh4, Myh11, Tpm2	Striated Muscle Contraction, Muscle contraction, RHO GTPases activate PAKs, Nephrin family interaction, Platelet activation, signaling and aggregation, Cell-Cell communication, Platelet degranulation, Signaling by Rho GTPases, Response to elevated platelet cytosolic Ca2+, Signaling by Rho GTPases, Miro GTPases and RHOBTB3	positive regulation of fast-twitch skeletal muscle fiber contraction, regulation of fast-twitch skeletal muscle fiber contraction, regulation of twitch skeletal muscle contraction, regulation of skeletal muscle contraction, regulation of muscle contraction, regulation of muscle system process, regulation of the force of skeletal muscle contraction, regulation of skeletal muscle contraction by chemo-mechanical energy conversion, muscle filament sliding, actin-myosin filament sliding	calcium-dependent ATPase activity, ATP-dependent activity, creatine kinase activity, phosphotransferase activity, nitrogenous group as acceptor, microfilament motor activity, cytoskeletal motor activity, actin filament binding, actin binding, cytoskeletal protein binding, protein binding	muscle thin filament tropomyosin, protein-containing complex, striated muscle thin filament, sarcomere, myofibril, contractile fiber, cytoplasm, intracellular anatomical structure, supramolecular fiber, supramolecular polymer
Metabolic stress response	Etfa, Hist1h1d, Gnai1, Gorasp2, Hspd1, Phgdh, Psma7, Hist1h1e, Rps11, Copb2, Actg1, Tpgs1, Uqcrc1, Sec23ip, Rdh14, Ppib, Rps26, Rplp0, H3f3a, Rps8, Eno1, Cers2, Pdia6, Tkt, Ywhaz, Actb, Ywhae, Idh1, Mdh2, Gnb2l1, Tubb5, Hist1h1c, Apoa1bp, Pdhb, Txn1, Hist2h3c2, Uqcrc2, Hnrnpm, Rps4x, Zdhhc14, Hist1h3g, Hist1h2bh, Hars, Canx, Mcm7, Zcchc14, Rps2, Ppox, Ruvbl1, Vdac3, Sucla2, Vdac1, Zdhhc13, Dpysl2, Gnb1, Rps3, Jph4, Olfr814, Tcp1, Hspa4, Eno3, Pgk1-rs7, March4, Pkm, C1qbp, Rpl13a, Eef1a1, Eef2, Hist1h4j, Vcp, Mcm3, Hsph1, Tpgs2, Pnp, Mrs2, Olfr559, Prdx2, Adh5, Gtf2h4, Ran, Rpl11, Pcna, Sdha, Msh4, Rpl30, Dlat, Puf60, Atp5o, Gnb2, Gm10116, Hspa8	Cellular responses to stress, Cellular responses to stimuli, Peptide chain elongation, Eukaryotic Translation Elongation, Nonsense Mediated Decay (NMD) independent of the Exon Junction Complex (EJC), Formation of a pool of free 40S subunits, Eukaryotic Translation Termination, Selenocysteine synthesis, Viral mRNA translation, Response of EIF2Ak4 (GCN2) to amino acid deficiency	positive regulation of deoxyribonuclease activity, positive regulation of nuclease activity, regulation of DNA metabolic process, regulation of transepithelial transport, DNA strand elongation involved in DNA replication, DNA strand elongation, cellular macromolecule metabolic process, cellular metabolic process, metabolic process, cellular process	phosphopyruvate hydratase activity, catalytic activity, structural constituent of postsynaptic actin cytoskeleton, structural molecule activity, porin activity, pyruvate dehydrogenase (NAD+) activity, pyruvate dehydrogenase [NAD(P)+] activity, pyruvate dehydrogenase activity, oxidoreductase activity, ADP binding	phosphopyruvate hydratase complex, catalytic complex, protein-containing complex, cytosol, cytoplasm, intracellular anatomical structure, cellular anatomical entity, mitochondrial pyruvate dehydrogenase complex, mitochondrial matrix, mitochondrion
Collagen and ECM biosynthesis	Tnc, Cilp, Col14a1, Col2a1, Serpinh1, Hapln1, Srpx, Col6a6, Comp, Dcn, Chad, Col5a2, Dpt, Col6a2, Col1a1, Susd1, Tnn, Htra1, Col6a1, Lum, Lamb2, Col1a2, Fbn1, Sparc, Col9a1, Lamc1, Bgn, Tgfbi, Col12a1, Thbs4, Acan, Postn, Cspg4, Col3a1, Prelp, Col4a1, Clec3a, Col10a1	Assembly of collagen fibrils and other multimeric structures, Collagen formation, Collagen biosynthesis and modifying enzymes, Collagen degradation, ECM proteoglycans, Extracellular matrix organization, Collagen chain trimerization, Integrin cell surface interactions, Degradation of the extracellular matrix, Non-integrin membrane-ECM interactions	peptide cross-linking via chondroitin 4-sulfate glycosaminoglycan, peptide cross-linking, proteoglycan metabolic process, collagen biosynthetic process, collagen fibril organization, supramolecular fiber organization, cellular component organization, cellular component organization or biogenesis, extracellular matrix organization, extracellular structure organization	platelet-derived growth factor binding, growth factor binding, extracellular matrix structural constituent conferring compression resistance, extracellular matrix structural constituent, structural molecule activity, extracellular matrix structural constituent conferring tensile strength, collagen binding, protein-containing complex binding, extracellular matrix binding, fibronectin binding	collagen type I trimer, fibrillar collagen trimer, banded collagen fibril, supramolecular fiber, supramolecular polymer, supramolecular complex, complex of collagen trimers, collagen-containing extracellular matrix, extracellular matrix, external encapsulating structure

Table S5. Louvain community summary descriptors derived from overrepresented terms in Reactome pathway analysis and Gene Ontology analysis. Top-ten GO terms of each category shown.

YOUNG					
**Accession**	**Protein name**	**Protein length (residues)**	**Net charge**	**ΔΔG (kT)**	**PTM change (%/site)**
Q61282|PGCA_MOUSE	Aggrecan core protein	2132	192.4	11.7	5.5
Q61554|FBN1_MOUSE	Fibrillin-1	2873	115	5.8	-25.6
Q9Z1T2|TSP4_MOUSE	Thrombospondin-4	963	61.4	5.7	33.3
P58771|TPM1_MOUSE	Tropomyosin alpha-1 chain	284	26.2	4.3	-20.0
P28293|CATG_MOUSE	Cathepsin G	261	19.6	3.6	50.0
P11247|PERM_MOUSE	Myeloperoxidase	718	31.2	3.5	0.7
Q80X19|COEA1_MOUSE	Collagen alpha-1(XIV) chain	1797	48.2	3.2	50.0
Q9DB20|ATPO_MOUSE	ATP synthase subunit O, mitochondrial	213	15.4	3.0	-16.7
P99024|TBB5_MOUSE	Tubulin beta-5 chain	444	21	2.7	5.0
P62889|RL30_MOUSE	60S ribosomal protein L30	115	10.8	2.7	50.0
Q61879|MYH10_MOUSE	Myosin-10	1976	43.4	2.7	-8.3
P08071|TRFL_MOUSE	Lactotransferrin	707	23.2	2.7	8.1
Q7TPR4|ACTN1_MOUSE	Alpha-actinin-1	892	28.2	2.6	16.7
O55029|COPB2_MOUSE	Coatomer subunit beta	905	26.8	2.5	33.3
**AGED**					
**Accession**	**Protein name**	**Protein length (residues)**	**Net charge**	**ΔΔG (kT)**	**PTM change (%/site)**
Q61282|PGCA_MOUSE	Aggrecan core protein	2132	192.4	11.7	-1.7
P14211|CALR_MOUSE	Calreticulin	416	52.2	7.3	-33.3
P35564|CALX_MOUSE	Calnexin	591	54.4	6.5	-33.3
Q61554|FBN1_MOUSE	Fibrillin-1	2873	115	5.8	-25.0
Q80YX1|TENA_MOUSE	Tenascin	2110	94.6	5.7	-37.5
Q9Z1T2|TSP4_MOUSE	Thrombospondin-4	963	61.4	5.7	-50.0
P84228|H32_MOUSE	Histone H3.2	136	20.8	4.8	-9.1
P62806|H4_MOUSE	Histone H4	103	18.8	4.8	-8.2
P58771|TPM1_MOUSE	Tropomyosin alpha-1 chain	284	26.2	4.3	6.7
P28293|CATG_MOUSE	Cathepsin G	261	19.6	3.6	-33.3
P11247|PERM_MOUSE	Myeloperoxidase	718	31.2	3.5	3.6
Q8VHY0|CSPG4_MOUSE	Chondroitin sulfate proteoglycan 4	2327	61.6	3.4	-100.0
O55226|CHAD_MOUSE	Chondroadherin	358	19.8	3.2	-50.0
Q80X19|COEA1_MOUSE	Collagen alpha-1(XIV) chain	1797	48.2	3.2	-66.7
P62259|1433E_MOUSE	14-3-3 protein epsilon	255	17.8	3.0	-16.7
O08638|MYH11_MOUSE	Myosin-11	1972	43.4	2.7	-4.2
P08071|TRFL_MOUSE	Lactotransferrin	707	23.2	2.7	16.2
P57780|ACTN4_MOUSE	Alpha-actinin-4	912	29	2.6	-8.3
Q7TPR4|ACTN1_MOUSE	Alpha-actinin-1	892	28.2	2.6	19.3

Table S6. Articular joint proteins with detected PTMs drawn from the largest quartile of folding free energy stability change upon oxidative destabilization, including outliers not plotted in Figure 4.

SUPPLEMENTARY INFORMATION REFERENCES

Akasaki, Y, Reixach, N, Matsuzaki, T, Alvarez-Garcia, O, Olmer, M, Iwamoto, Y, Buxbaum, JN, Lotz, MK. (2015). Transthyretin deposition in articular cartilage: a novel mechanism in the pathogenesis of osteoarthritis. *Arthritis & Rheumatology*, 2097-2107.

Barreto, G, Soininen, A, Ylinen, P, Sandelin, J, Konttinen, YT, Nordstrom, DC, Eklund, KK. (2015). Soluble biglycan: a potential mediator of cartilage degradation in osteoarthritis. *Arthritis Research and Therapy*, 17(1), 1-15.

Bastian, M, Heymann, S, Jacomy, M. (2009). Gephi: an open source software for exploring and manipulating networks. Proceedings of the international AAAI conference on web and social media 3(1), 361-362.

Blondel, VD, Guillaume, J-L, Lambiotte, R, Lefebvre, E. (2008). Fast unfolding of communities in large networks. *Journal of statistical mechanics: theory and experiment*, 2008(10), P10008.

Bomalaski, JS, Clark, MA. (1993). Phospholipase A2 and arthritis. *Arthritis and rheumatism*, 36(2), 190-198.

Bonacich, P. (1971). Factoring and Weighing Approaches to Clique Identification. *Journal of Mathematical Sociology*, 92, 1170-1182.

Dalle-Donne, I, Rossi, R, Giustarini, D, Milzani, A, Colombo, R. (2003). Protein carbonyl groups as biomarkers of oxidative stress. *Clinica chimica acta*, 329(1-2), 23-38.

Dore, E, Boilard, E. (2019). Roles of secreted phospholipase A2 group IIA in inflammation and host defense. *Biochimica et Biophysica Acta (BBA)-Molecular and Cell Biology of Lipids*, 1864(6), 789-802.

Consortium, GO. (2019). The gene ontology resource: 20 years and still GOing strong. *Nucleic acids research*, 47(D1), D330-D338.

Genetos, DC, Wong, A, Weber, TJ, Karin, NJ, Yellowley, CE. (2014). Impaired osteoblast differentiation in annexin A2-and-A5-deficient cells. *PloS one*, 9(9), e107482.

Gillespie, M, Jassal, B, Stephan, R, Milacic, M, Rothfels, K, Senff-Ribeiro, A, Griss, J, Sevilla, C, Matthews, L, Gong, C. (2022). The reactome pathway knowledgebase 2022. *Nucleic acids research*, 50(D1), D687-D692.

Griveau, A, Wiel, C, Le Calvé, B, Ziegler, DV, Djebali, S, Warnier, M, Martin, N, Marvel, J, Vindrieux, D, Bergo, MO, Bernard, D. (2018). Targeting the phospholipase A2 receptor ameliorates premature aging phenotypes. *Aging Cell*, 17(6), e12835.

Hagberg, A, Swart, P, S Chult, D. (2008). Exploring network structure, dynamics, and function using NetworkX., LA-UR.

Han, E-S, Muller, FL, Pérez, VI, Qi, W, Liang, H, Xi, L, Fu, C, Doyle, E, Hickey, M, Cornell, J. (2008). The in vivo gene expression signature of oxidative stress. *Physiological genomics*, 34(1), 112-126.

Hosseininia, S, Weis, MA, Rai, J, Kim, L, Funk, S, Dahlberg, LE, Eyre, DR. (2016). Evidence for enhanced collagen type III deposition focally in the territorial matrix of osteoarthritic hip articular cartilage. *Osteoarthritis and cartilage*, 24(6), 1029-1035.

Huang, S, Thomsson, KA, Jin, C, Alweddi, S, Struglics, A, Rolfson, O, Bjorkman, LI, Kalamajski, S, Schmidt, TA, Jay, GD, Krawetz, R, Karlsson, NG, Eisler, T. (2020). Cathepsin g degrades both glycosylated and unglycosylated regions of lubricin, a synovial mucin. *Scientific reports*, 10(1), 4125.

Janelle-Montcalm, A, Boileau, C, Poirier, F, Pelletier, J-P, Guévremont, M, Duval, N, Martel-Pelletier, J, Reboul, P. (2007). Extracellular localization of galectin-3 has a deleterious role in joint tissues. *Arthritis research & therapy*, 9(1), 1-9.

Jeon, OH, Wilson, DR, Clement, CC, Rathod, S, Cherry, C, Powell, B, Lee, Z, Khalil, AM, Green, JJ, Campisi, J, Santambrogio, L, Witwer, KW, Elisseeff, JH. (2019). Senescence cell–associated extracellular vesicles serve as osteoarthritis disease and therapeutic markers. *Jci Insight*.

Jeon, YJ, Kim, DH, Jung, H, Chung, SJ, Chi, SW, Cho, S, Lee, SC, Park, BC, Park, SG, Bae, KH. (2010). Annexin A4 interacts with the NF-κB p50 subunit and modulates NF-κB transcriptional activity in a Ca 2+-dependent manner. *Cellular and molecular life sciences*, 67, 2271-2281.

Kaneva, MK, Muley, MM, Krustev, E, Reid, AR, Souza, PR, Dell'Accio, F, McDougall, JJ, Perretti, M. (2021). Alpha-1-antitrypsin reduces inflammation and exerts chondroprotection in arthritis. *The FASEB Journal*, 35(5), e21471.

Karlsson, C, Dehne, T, Lindahl, A, Brittberg, M, Pruss, A, Sittinger, M, Ringe, J. (2010). Genome-wide expression profiling reveals new candidate genes associated with osteoarthritis. *Osteoarthritis and cartilage*, 18(4), 581-592.

Kim, HJ, Kim, KS, Kim, SH, Baek, S-H, Kim, HY, Lee, C, Kim, J-R. (2009). Induction of cellular senescence by secretory phospholipase A2 in human dermal fibroblasts through an ROS-mediated p53 pathway. *Journals of Gerontology Series A: Biomedical Sciences and Medical Sciences*, 64(3), 351-362.

Kirsch, T, Minashima, T, Campbell, K, Zhang, Y. (2013). Annexin A6: a novel therapeutic target for the treatment of osteoarthritis. *Osteoarthritis and cartilage*, 21, S226-S227.

Krügel, J, Sadowski, B, Miosge, N. (2008). Nidogen-1 and nidogen-2 in healthy human cartilage and in late-stage osteoarthritis cartilage. *Arthritis & Rheumatism*, 58(5), 1422-1432.

Lex, A, Gehlenborg, N, Strobelt, H, Vuillemot, R, Pfister, H. (2014). UpSet: visualization of intersecting sets. *IEEE transactions on visualization and computer graphics*, 20(12), 1983-1992.

Lorenzo, P, Bayliss, MT, Heinegård, D. (1998). A novel cartilage protein (CILP) present in the mid-zone of human articular cartilage increases with age. *Journal of Biological Chemistry*, 273(36), 23463-23468.

Lu, J, Lian, G, Lenkinski, R, De Grand, A, Vaid, RR, Bryce, T, Stasenko, M, Boskey, A, Walsh, C, Sheen, V. (2007). Filamin B mutations cause chondrocyte defects in skeletal development. *Human molecular genetics*, 16(14), 1661-1675.

Maly, K, Schaible, I, Riegger, J, Brenner, RE, Meurer, A, Zaucke, F. (2019). The expression of thrombospondin-4 correlates with disease severity in osteoarthritic knee cartilage. *International journal of molecular sciences*, 20(2), 447.

Miosge, N, Hartmann, M, Maelicke, C, Herken, R. (2004). Expression of collagen type I and type II in consecutive stages of human osteoarthritis. *Histochemistry and cell biology*, 122, 229-236.

Ozkan, H, Willcockson, HH, Esterellas, A, Le, AM, Longobardi, L. (2020). Fetuin-A as critical factor to maintain joint and muscle integrity after injury. *Osteoarthritis and Cartilage*, 28, S191.

Perez-Riverol, Y, Bai, J, Bandla, C, García-Seisdedos, D, Hewapathirana, S, Kamatchinathan, S, Kundu, DJ, Prakash, A, Frericks-Zipper, A, Eisenacher, M. (2022). The PRIDE database resources in 2022: a hub for mass spectrometry-based proteomics evidences. *Nucleic acids research*, 50(D1), D543-D552.

Pruzanski, W, Vadas, P, Stefanski, E, Urowitz, MB. (1985). Phospholipase A2 activity in sera and synovial fluids in rheumatoid arthritis and osteoarthritis. Its possible role as a proinflammatory enzyme. *The Journal of rheumatology*, 12(2), 211-216.

Reesink, HL, Bonnevie, ED, Liu, S, Shurer, CR, Hollander, MJ, Bonassar, LJ, Nixon, AJ. (2016). Galectin-3 binds to lubricin and reinforces the lubricating boundary layer of articular cartilage. *Scientific reports*, 6(1), 1-10.

Rigoglou, S, Papavassiliou, AG. (2013). The NF-κB signalling pathway in osteoarthritis. *The international journal of biochemistry & cell biology*, 45(11), 2580-2584.

Scharf, B, Clement, C, Yodmuang, S, Urbanska, AM, Suadicani, SO, Aphkhazava, D, Thi, MM, Perino, G, Hardin, J, Cobelli, N, Vunjak-Novakovic, G, Santambrogio, L. (2013). Age-Related Carbonylation of Fibrocartilage Structural Proteins Drives Tissue Degenerative Modification. *Chemistry & Biology*, 20(7), 922-934.

Szklarczyk, D, Gable, AL, Lyon, D, Junge, A, Wyder, S, Huerta-Cepas, J, Simonovic, M, Doncheva, NT, Morris, JH, Bork, P. (2019). STRING v11: protein–protein association

networks with increased coverage, supporting functional discovery in genome-wide experimental datasets. *Nucleic acids research*, 47(D1), D607-D613.

Tsuruha, J, Masuko-Hongo, K, Kato, T, Sakata, M, Nakamura, H, Nishioka, K. (2001). Implication of cartilage intermediate layer protein in cartilage destruction in subsets of patients with osteoarthritis and rheumatoid arthritis. *Arthritis & Rheumatism*, 44(4), 838-845.

Uhlén, M, Fagerberg, L, Hallström, BM, Lindskog, C, Oksvold, P, Mardinoglu, A, Sivertsson, Å, Kampf, C, Sjöstedt, E, Asplund, A. (2015). Tissue-based map of the human proteome. *Science*, 347(6220), 1260419.

Wang, L, Zhai, Q, Zhao, P, Xiang, X, Zhang, X, Tian, W, Li, T. (2018). Functional analysis of p.Ala253_Leu254insAsn mutation in PLS3 responsible for X-linked osteoporosis. *Clinical genetics*, 93(1), 178-181.

Waskom, ML. (2021). Seaborn: statistical data visualization. *Journal of Open Source Software*, 6(60), 3021.

Wu, W, Pasierb, M. (2011). Kinins can Stimulate the Upregulation and Production of MMPs in Osteoarthitic Cartilage. *ORS 2011 Annual Meeting*, 266.

Youlten, SE, Kemp, JP, Logan, JG, Ghirardello, EJ, Sergio, CM, Dack, MRG, Guilfoyle, SE, Leitch, VD, Butterfield, NC, Komla-Ebri, D. (2021). Osteocyte transcriptome mapping identifies a molecular landscape controlling skeletal homeostasis and susceptibility to skeletal disease. *Nature Communications*, 12(1), 2444.
